# Detection of Estrogen Receptor Alpha and Assessment of Fulvestrant Activity in MCF-7 Tumor Spheroids Using Microfluidics and SERS

**DOI:** 10.1021/acs.analchem.1c00188

**Published:** 2021-04-02

**Authors:** Anastasia Kapara, Karla A. Findlay Paterson, Valerie G. Brunton, Duncan Graham, Michele Zagnoni, Karen Faulds

**Affiliations:** † Centre for Molecular Nanometrology, Department of Pure and Applied Chemistry, Technology and Innovation Centre, University of Strathclyde, 99 George Street, Glasgow G1 1RD, UK; ‡ MRC Institute of Genetics and Molecular Medicine, Edinburgh Cancer Research UK Centre, University of Edinburgh, Western General Hospital, Crewe Road South, Edinburgh EH4 2XU, UK; § Centre for Microsystems and Photonics, Department of Electronic and Electrical Engineering, University of Strathclyde, 204 George Street, Glasgow G1 1XW, UK

## Abstract

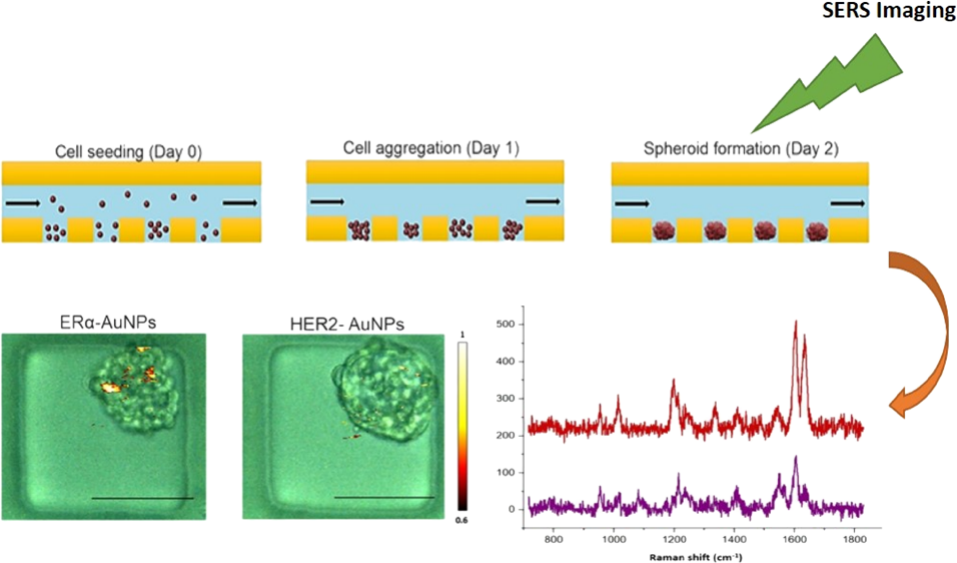

Breast cancer is one of the leading causes of cancer death in women. Novel in vitro tools that integrate three-dimensional (3D) tumor models with highly sensitive chemical reporters can provide useful information to aid biological characterization of cancer phenotype and understanding of drug activity. The combination of surface-enhanced Raman scattering (SERS) techniques with microfluidic technologies offers new opportunities for highly selective, specific, and multiplexed nanoparticle-based assays. Here, we explored the use of functionalized nanoparticles for the detection of estrogen receptor alpha (ERα) expression in a 3D tumor model, using the ERα-positive human breast cancer cell line MCF-7. This approach was used to compare targeted versus nontargeted nanoparticle interactions with the tumor model to better understand whether targeted nanotags are required to efficiently target ERα. Mixtures of targeted anti-ERα antibody-functionalized nanotags (ERα-AuNPs) and nontargeted (against ERα) anti-human epidermal growth factor receptor 2 (HER2) antibody-functionalized nanotags (HER2-AuNPs), with different Raman reporters with a similar SERS signal intensity, were incubated with MCF-7 spheroids in microfluidic devices and spectroscopically analyzed using SERS. MCF-7 cells express high levels of ERα and no detectable levels of HER2. 2D and 3D SERS measurements confirmed the strong targeting effect of ERα-AuNP nanotags to the MCF-7 spheroids in contrast to HER2-AuNPs (63% signal reduction). Moreover, 3D SERS measurements confirmed the differentiation between the targeted and the nontargeted nanotags. Finally, we demonstrated how nanotag uptake by MCF-7 spheroids was affected by the drug fulvestrant, the first-in-class approved selective estrogen receptor degrader (SERD). These results illustrate the potential of using SERS and microfluidics as a powerful in vitro platform for the characterization of 3D tumor models and the investigation of SERD activity.

Breast cancer is a major health issue among women worldwide.^1,2^ In the UK, one person is diagnosed with breast cancer every 10 min and one in eight women will develop breast cancer at some point in their lives.^3^ Various studies have shown that breast cancer proliferation and metastasis are highly affected by the cancer cellular and physical microenvironment.^4−6^ A limitation of cell-based studies for breast cancer is that the majority of the characterization and development of new therapeutic agents are conducted in two-dimensional (2D) monolayer cell cultures.^7^ Therefore, cellular processes, such as drug transport and cell–cell and/or cell–matrix interactions, are not taken into consideration.^8,9^ Studies have shown that 2D breast cancer cell cultures have different behaviors, gene expression, and, usually, higher sensitivity to anti-cancer drugs than three-dimensional (3D) models.^10,11^ Significantly, many drug compounds that have been found to be efficient in 2D cultures have failed in clinical trials.^12,13^ These findings justify the need for using 3D in vitro tumor models to better recapitulate certain aspects of the in vivo breast cancer microenvironment.

Microfluidic technologies offer a powerful tool for the creation of 3D cancer models (e.g., spheroids) and in vitro mechanistic studies.^14,15^ The technology is an excellent tool to bridge the gap between 2D monolayer cultures and animal models, offering cost-effective solutions for miniaturized yet high throughput assays with high accuracy, faster analysis, and potential for automation.^16−19^

Importantly, microfluidics can be combined with analytical spectroscopic methods, such as fluorescence microscopy^20−22^ or surface-enhanced Raman spectroscopy (SERS).^23^ For instance, the combination of SERS with microfluidic devices has been applied to rapid analysis of food contaminants,^24^ multiplex recognition of interleukins from blood plasma,^25^ and detection of prostate cancer biomarkers.^26^ Aberasturi et al. focused on using microfluidic devices and 3D SERS imaging to distinguish unlabeled and nanotag-labeled fibroblast cells to mimic different cell populations within a tissue.^27^ Moreover, Altunbek et al. used SERS and the hanging drop method to monitor the cellular response to drug exposure.^28^

SERS offers signal enhancement factors of 10^4^–10^8^ in comparison to conventional Raman by adsorbing a molecule of interest onto a roughened metal surface, such as colloidal suspensions of gold and silver nanoparticles.^29−31^ SERS is a noninvasive technique, compared to other destructive analytical methods such as transition electron microscopy (TEM), that offers high specificity, selectivity, and multiplexed capabilities due to the sharp fingerprint spectra produced.^32,33^ Additionally, fluorescence imaging is prone to photobleaching making 3D imaging exceptionally challenging since bleaching can compromise the definition of 3D structures leading to false results. Moreover, fluorescence, in contrast to SERS, generates a broad emission band making the detection of multiple components within the same sample challenging in a 3D structure. Recently, there have been significant developments in using SERS for cancer imaging^34−36^ and drug screening.^37,38^

The enhanced permeability and retention (EPR) effect has been the main reason behind the high enthusiasm for the development of nanoparticles in cancer research. The EPR effect is the mechanism by which nanoparticles passively accumulate at a tumor sites.^39^ Although the EPR effect provides an advantage toward the nanoparticles since they remain in the tumor site for imaging for relatively long periods,^40^ its function can be unpredictable and highly heterogeneous in preclinical animal models with doubts over how valid this effect is in humans.^41−43^ Therefore, active targeting of nanoparticles, using antibodies and peptides, has been developed to efficiently target the nanoparticles to the tumor site.^44,45^ Antibodies can be used as targeting agents attached to the nanoparticles since the concentration of certain proteins expressed in the body can be used as a biomarker of cell function that indicates the presence of a pathological condition. The targeted nanoparticles are therefore bound to the tumor site based on their specific biological activity.^40^ Studies have shown that the targeted nanoparticles increase the number of nanomaterials taken up by the tumor cells^46^ and the total accumulation at the tumor site, thus minimizing nonspecific effects.^47^ Smith et al. have shown that ligand-targeted nanomaterials accumulated more in tumors and bound to individual tumor cells than the nonligand targeted nanomaterials that were cleared during the experimental time.^42^

In this study, we developed a novel assay that combines microfluidic and SERS techniques for tumor identification, phenotype characterization, and assessment of drug activity in 3D breast cancer spheroids. More importantly, we used targeted gold nanoparticles (AuNPs) functionalized with anti-estrogen receptor alpha antibodies (ERα-AuNPs) and nontargeted (against ERα) nanoparticles functionalized with anti-human epidermal growth factor antibodies (HER2-AuNPs) for the characterization of ERα overexpressing MCF-7 spheroids using SERS. This approach gave us a great insight into the benefits of using targeted nanotags versus nontargeted ones in a 3D environment for the characterization of ERα cancer phenotype. In addition, we investigated the effects of fulvestrant activity, a commercially available selective estrogen receptor degrader (SERD). This proof-of-concept work opens up opportunities for using 3D models, microfluidics, and SERS to investigate in a miniaturized and scalable manner the drug response of advanced in vitro models to preclinical compounds.

## Experimental Section

### Materials

Anti-estrogen receptor alpha (ERα) antibody (ab16660) and Anti-Erb2 (HER2) antibody (ab16899) were purchased from Abcam (330 Cambridge Science Park, Cambridge, CB4 0FL, UK). Anti-mouse IgG HRP-linked antibody (7076S) and anti-rabbit IgG HRP-linked antibody (7074S) were purchased from Cell Signalling Technology (Hamilton House, Mabledon Place, London, WC1H 9BB, UK). Sodium tetrachloroaurate dihydrate, (*N*-(3-dimethylaminopropyl)-*N*′-ethylcarbodiimide hydrochloride) (EDC), *N*-hydroxysulfosuccinimide sodium salt (NHS), poly(ethylene glycol) 2-mercaptoethyl ether acetic acid (HS-PEG5000-COOH), dynasore hydrate, 1,2-bis(4-pyridyl) ethylene (BPE), 4-(2-hydroxyethyl)-1-piperazineethancesulfonic acid (HEPES), 2-(*N*-morpholino) ethanesulfonic acid (MES), LIVE/DEAD Viability/Cytotoxicity Assay Kit diacetate (FDA)-propidium iodide (PI), and Synperonic F108 were obtained from Sigma-Aldrich Ltd. (The Old Brickyard, New Road, Gillingham, Dorset, SP8 4XT, UK). The LIVE/DEAD Viability/Cytotoxicity Assay Kit was purchased from ThermoFisher Scientific (3 Fountain Drive, Inchinnan, Renfrew PA4 9RF, UK). Milli-Q deionized water was used after purification using a Milli-Q purification system. All glassware was cleaned in aqua regia (3 HCl:1 HNO_3_).

### Device Design Preparation

Multilayered microfluidic devices were produced using standard soft lithography techniques and used for culturing spheroids, following established protocols. Each device consisted of a microfluidic channel connected by two open wells. Each channel hosted four arrays of 64 square microwells (150 × 150 × 150 μm^3^), as previously reported.^48^ Briefly, a 10:1 ratio of the polydimethylsiloxane (PDMS) prepolymer (Sylgard 184, Dow Corning) to the curing agent was mixed and dispensed onto patterned silicon wafers. The wafers were degassed and subsequently incubated at 85 °C for a minimum of 3 h to allow curing of the PDMS solution. PDMS layers were then cut from the wafers, and open wells were formed using a 4 mm surgical biopsy punch (Miltex). The PDMS layers were cleaned and treated with oxygen plasma (Pico plasma cleaner, Diener electronic) to permanently bond the layers together, forming a microfluidic device. Devices were then stored overnight at 85 °C and exposed a second time to oxygen plasma before injecting a 1% solution of Synperonic F108 (Sigma Aldrich), creating ultra-low-adhesion conditions. Subsequently, devices were washed using phosphate-buffered saline (PBS) and Roswell Park Memorial Institute 1640 culture medium (RPMI). Devices were stored at 37 °C and 5% CO_2_ in a humidified incubator prior to cell seeding. Cells were seeded into devices at a concentration of 5 × 10^6^ cells/mL to form spheroids as previously reported.^48^ At least 32 spheroids per conditions were analyzed. The medium was exchanged every 48 h.

### Nanoparticle Synthesis and Functionalization of ERα-AuNP and HER2-AuNP Nanotags

Bare AuNPs were synthesized by standard citrate reduction of gold.^49^ The functionalization, characterization, and stability of ERα-AuNPs SERS nanotags have been previously reported by our group.^50^ Briefly, anti-ERα antibodies were attached to the AuNP gold surface via carbodiimide cross-linking chemistry. The coupling chemistry was achieved after the attachment of the 1,2-bis(4-pyridyl) ethylene (BPE) Raman reporter to the AuNP surface. For anti-HER2 functionalization, 10 μL of 4-(1*H*-pyrazol-4-yl)pyridine (PPY) (0.1 μM) was added to bare AuNPs (0.03 nM, 990 μL) and the solution was incubated on a shaker plate for 30 min followed by centrifugation at 6000 rpm for 20 min. The solution of EDC-NHS-PEG5000-mAb was added dropwise to the pelleted PPY-AuNPs. The nanotags were incubated on a shaker plate for 3 h. The free protein was removed by centrifugation at 6000 rpm for 10 min and was used for protein estimation analysis. The nanotags did not demonstrate any aggregation and maintained their strong and characteristic SERS signals.

### Nanoparticle Characterization

Extinction spectra were measured using an Agilent Cary 60 UV–Visible (UV–vis) spectrophotometer with Win UV scan V.2.00 software. The instrument was allowed to equilibrate to RT before using poly(methyl methacrylate) (PMMA) disposable plastic micro cuvettes with 500 μL sample volumes to scan wavelengths of 300–800 nm. Where required, samples were diluted to give extinction values of less than 1 to adhere to the Beer–Lambert law, to allow calculation of the concentration of AuNPs. Dynamic light scattering (DLS) and zeta potential were measured using a Malvern Zetasizer Nano ZS with 800 μL of the sample in a PMMA disposable microcuvette with Zetasizer μV and APS v.6.20 software. Polystyrene latex beads (40 nm) were used as a standard to validate the calibration of the system before running samples. Measurements were taken in triplicate. For the solution measurements of the nanotags, SERS analysis was carried out using a Snowy Range CBEx 2.0 handheld Raman spectrometer (Snowy Range Instruments, Laramie, WY, USA) equipped with a 638 nm laser with a maximum laser power of 40 mW. Samples were placed into glass vials for analysis. The sample volumes were 600 μL, and spectra were collected using 100% laser power at the sample with a 0.05 s accumulation time. The software used to acquire spectra was Peak 1.1.112.

### 2D Breast Cancer Cell Culture

MCF-7 cells (ATCC HTB-22) were obtained from American Type Culture Collection (ATCC) (Queens Road, Teddington, Middlesex, TW11 0LY, UK). The human breast cancer cells were cultured in a Rosewell Park Memorial Institute medium (RPMI 1640) supplemented with 1% penicillin/streptomycin (10,000 units per mL), 1% fungizone, and 10% heat-inactivated fetal bovine serum (FBS). Cells were incubated at 37 °C and 5% CO_2_ in a humidified incubator. Cells at a confluence of ca. 90% growing in a T175 flask were trypsinized and resuspended in the RMPI medium.

### Nanotag Loading and Fulvestrant Treatment in Microfluidic Devices

For the purposes of this study, the initial stock of fulvestrant was made in DMSO and stored at 4 °C. According to the supplier’s instructions, the solubility of fulvestrant in water is 9.53 × 10^–3^ mg/mL while in DMSO it is 20 mg/mL at 25 °C. For the dilution of fulvestrant stock, water was used to ensure that the residual amount of organic solvent was insignificant and had no physiological effects on our models. Immediately prior to injection into devices, fulvestrant solution was diluted in RPMI media to the desired concentration. Spheroids were exposed to fulvestrant solution (1 and 10 μΜ) on the third day of culture in the microfluidic devices and incubated for 24 h at 37 °C and 5% CO_2_. This was followed by the removal of the drug and the addition of nanotags: ERα-AuNPs (60 pM), HER2-AuNPs (60 pM), or a mixture of ERα + HER2-AuNPs (60 pM). Nanotags were gently pipetted up and down prior to injection into devices, ensuring a flow of the nanotags through the entirety of the microfluidic channel. After a 2 h incubation period, nanotags were removed, and the channels were washed twice with PBS to remove any unbound nanotags. Control experiments were performed for each set of experiments.

### Cell Viability Studies in Microfluidic Devices

To determine spheroid viability throughout the culture period, staining of spheroids was performed at several time points: immediately after nanotag exposure (day 4), 3 days after nanotag exposure (day 7), and 6 days after nanotag exposure (day 10). Spheroids were stained with 8 μg/mL fluorescein diacetate (FDA) and 20 μg/mL propidium iodide (PI). The staining solution was added to the devices and then incubated for 30 min. PBS was then used to wash excess staining solution and was added a second time prior to imaging.

### Microscopy and Image Analysis

Spheroids were imaged via bright-field microscopy using an inverted microscope (Axio Observer Z1, Zeiss) connected to an Orca Flash 4.0 camera (Hamamatsu). Images were collected every second day and before and after drug treatment and nanoparticle exposure. Image analysis was performed using ZEN Blue, Fiji, and Matlab to estimate the spheroid area and perimeter. The viable fraction (Vf), a parameter used to quantify spheroid health, was calculated for each spheroid as the ratio of FDA stain area over the bright-field area on the day prior to drug administration, as previously reported.^48^ Spheroids possessing a Vf ≥ 1 were considered to have been unaltered by exposure to nanotags or fulvestrant treatment since they had either remained the same size or increased in size over the culture period. In contrast, spheroids with a Vf < 1 were regarded as unhealthy or as having been negatively affected due to administration of the nanotags or fulvestrant. The shape factor (Sf) of a spheroid, a marker of spheroid disaggregation, was also used as an assessment of its health, as previously described.^48^

### SERS Cell Mapping

A Renishaw InVia Raman confocal microscope (Renishaw, Wolton-under-Edge, U.K.) was used to generate 2D and 3D SERS data. For SERS mapping, the microfluidic devices were positioned upside down to allow scanning under an upright confocal microscope. This process resulted in the translocation of spheroids toward the corner of the device microwells without affecting their integrity. 2D SERS maps were collected using edge Streamline HR high confocality mode with 3 μm spatial resolution in the *x* and *y* directions. 3D SERS maps were collected using edge Streamline HR high confocality mode with a 3 μm step size resolution in the *x* and *y* directions and 4 μm between *z*-stacks. A 20× objective (0.40 NA) was used on the samples with a laser power of 12 mW (100% power) at the sample, from a HeNe 633 nm excitation source with a 0.1 s acquisition time per point, and a 1200 lines/mm grating in high confocality mode. A Windows-based Raman Environment (WiRE - Renishaw plc) 4.4 software package was used to preprocess the data by using their proprietary nearest neighbor, then width of feature cosmic ray removal, and baseline subtraction features. The image was generated using direct classical least squares analysis (DCLS) based on a BPE or PPY reference SERS spectrum whereby a false color was generated only when there was a good spectral fit between the reference and the collected spectra.

### Statistical Analysis

Statistical analysis was carried out with GraphPad Prism 8.1.2 (GraphPad Software, Inc., San Diego, CA). Student’s *t*-test was used for comparison of two variables, and one-way analysis of variance (ANOVA) with post-hoc Tukey’s test was used for comparison of three or more groups. Differences between groups were considered to be significant at a *P* value of <0.05.

## Results and Discussion

### Synthesis and Characterization of ERα-AuNPs and HER2-AuNPs

For the nanotag design, anti-ERα (ERα-AuNPs) or anti-HER2 (HER2-AuNPs) antibodies were covalently attached to the surface of 50 nm gold nanoparticles using the EDC-NHS coupling reaction.^51^ Poly(ethylene glycol) 2-mercaptoethyl ether acetic acid (HS-PEG5000-COOH) was added to the nanoparticle surface to avoid dissociation of the biomolecules, decrease toxic effects, and help the functionalization process. To confirm the successful functionalization, the nanotags were characterized using extinction spectroscopy, agarose electrophoresis, a lateral flow immunosorbent assay, and dynamic light scattering (DLS). The extinction spectra showed that there was a shift in LSPR when antibodies were added to the surface of the AuNPs (Figure S1A,B), indicating the successful attachment of the antibody to the metal surface. The nanotags did not demonstrate any indication of aggregation in the extinction spectra, suggesting that the AuNPs remained stable after the addition of the antibodies to their surface. The successful antibody functionalization was also confirmed with agarose gel electrophoresis since the PEG-AuNPs traveled further than the ERα-AuNPs and HER2-AuNPs, suggesting that the nanotags were of different size and/or charge (Figure S1C). The lateral flow immunosorbent assay (LFA) also showed that the antibodies were on the AuNP surface and that they remained active since a spot was observed on the detection zone of the nitrocellulose strip when the nanotags bound to their matching secondary IgG antibodies (anti-rabbit IgG for ERα and anti-mouse IgG for HER2). There was no spot observed when control PEG5000-AuNPs were tested with the anti-mouse IgG and anti-rabbit IgG, confirming the successful binding of the anti-ERα antibody and anti-HER2 to the AuNP surface (Figure S1D). Finally, DLS confirmed the successful antibody functionalization since the hydrodynamic diameter of the nanotags increased from 73.0 ± 1.0 to 80.3 ± 1.6 nm for ERα-AuNPs and to 79.8 ± 0.6 nm at pH 7.0 after the bioconjugation (Figure S1E,F). Therefore, the nanotag characterization confirmed that the AuNPs were successfully functionalized with ERα and HER2 antibodies that retained their bioactivities.

### Formation of MCF-7 Spheroids and Cell Viability Studies after Nanotag Incubation

**Figure 1:**
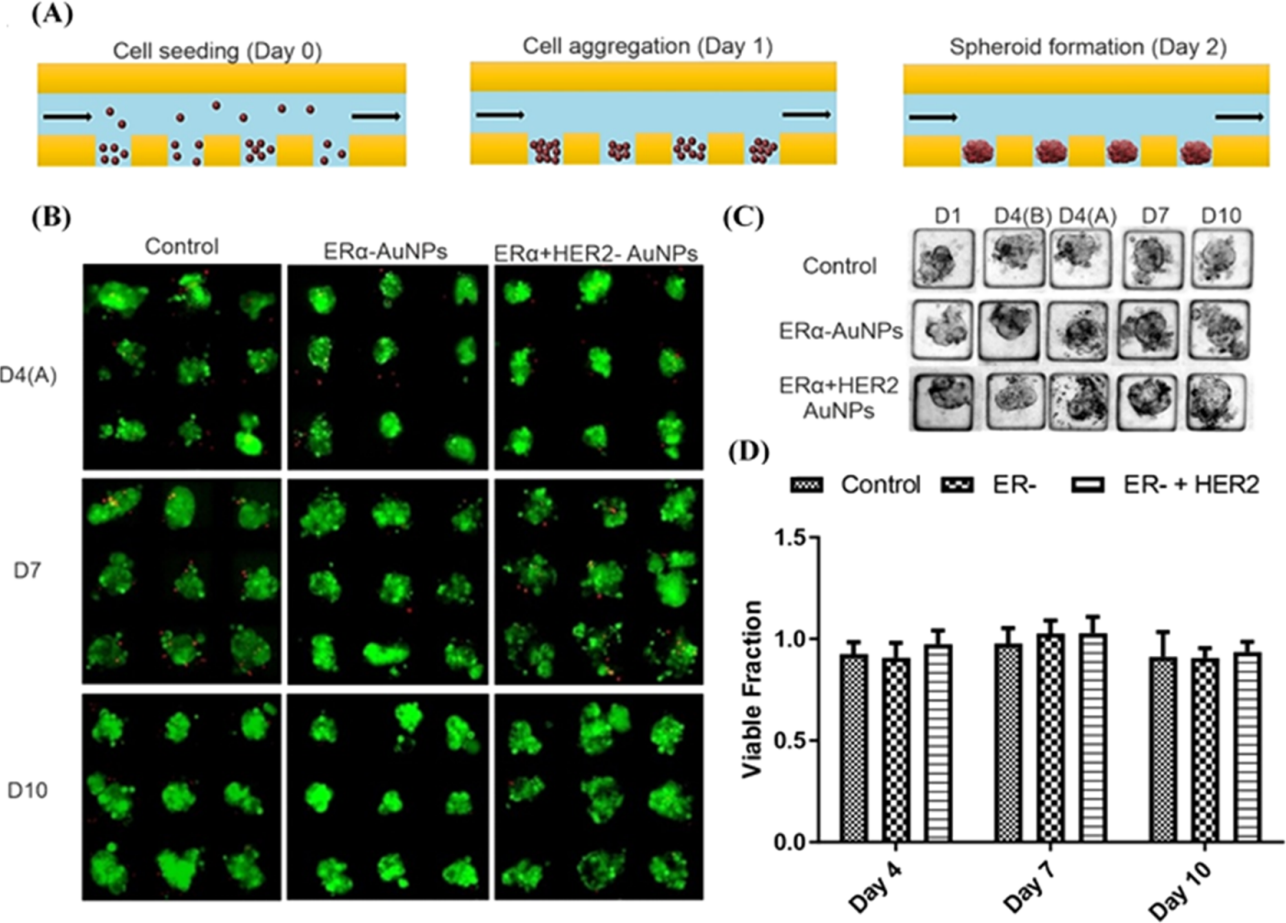
Effects of the ERα-AuNP and ERα + HER2-AuNP mixture on formation and viability of MCF-7 spheroids. (A) Schematic diagram showing the principle of formation of MCF-7 spheroids in the low-adhesion microfluidic device. (B) Representative images of spheroid viability at different time points. Viable cells appeared as green (fluorescein diacetate (FDA) staining), while nonviable cells appear as red (propidium iodide (PI) staining). (C) Bright-field images showing temporal evolution of MCF-7 spheroids cultured in a microfluidic device over a period of 10 days with ERα-AuNP and ERα + HER2-AuNP treatment. D1 = day 1 of cell seeding, D4(B) = day 4 of cell seeding (before the addition of nanotags), D4(A) = day 4 of cell seeding (after the addition of nanotags), D7 = day 7 of cell seeding, D10 = day 10 of cell seeding. (D) Bar plot of the viable fraction of the untreated spheroids, spheroids treated with ERα-AuNPs, and spheroids treated with the ERα + HER2-AuNP mixture. For the plots, each point was obtained from 32 spheroids. Error bars presented as mean ± S.D.

A suspension of ERα overexpressing MCF-7 cells was injected into microfluidic devices for spheroid formation. PDMS was used for the fabrication of microfluidic chips since it is a biocompatible, transparent polymer with low autofluorescence characteristics.^52,53^ Here, cells sedimented at the bottom of the device microwells, and due to low-adhesion conditions, spheroids were formed within 48 h of culture, and they were defined from their spherical and elliptical geometry (Figure 1A). MCF-7 cells express high levels of ERα and no detectable levels of HER2 (Figure S2). A Western blot confirmed that the MCF-7 cells used were positive for ERα but did not express any detectable levels of HER2 (Figure S2). An ERα-AuNP or the ER-α/HER2-AuNP mixture was incubated with the spheroids for 2 h. Spheroid viability was analyzed at different time points (day 4, day 7, and day 10) after the treatment with the ERα-AuNP or ERα + HER2-AuNP mixture. Results showed that the nanotags did not appear to have a toxic effect on the spheroids over the culture period (Figure 1B–D).

### Targeting Effect of ERα-AuNP Nanotags in MCF-7 Spheroids: 2D SERS Imaging

To determine the targeting effect of ERα-AuNPs in ERα-positive MCF-7 breast cancer spheroids, the spheroids were incubated with either the targeted ERα-AuNPs or with the nontargeted HER2-AuNPs. ERα-AuNP nanotags were labeled with a BPE Raman reporter, and HER2-AuNP nanotags were labeled with a PPY Raman reporter. Therefore, the targeted ERα-AuNP and the nontargeted HER2-AuNP nanotags had different reporters on their surface giving unique identificatory SERS signals. The concentrations of the reporters were balanced during the functionalization processes for ERα-AuNP and HER2-AuNP nanotags. This led to the nanotags having similar Raman intensities and, therefore, the same signal intensity per nanoparticle (Figure S3A). This normalization of the intensity per particle allowed the comparison in SERS intensity of the targeted and nontargeted nanoparticles. Before their addition to the spheroids, the ERα-AuNP (BPE Raman reporter) and HER2-AuNP (PPY Raman reporter) mixture in H_2_O was analyzed using SERS. Based on these SERS spectra, 1635 and 955 cm^–1^ were selected as representative peaks for ERα-AuNPs (BPE Raman reporter) and HER2-AuNPs (PPY Raman reporter), respectively (Figure S3B). The spectra from the two reporters confirmed that they have unique peaks to identify the ERα-AuNP and HER2-AuNP locations and targeting when both were present in the spheroids.

**Figure 2:**
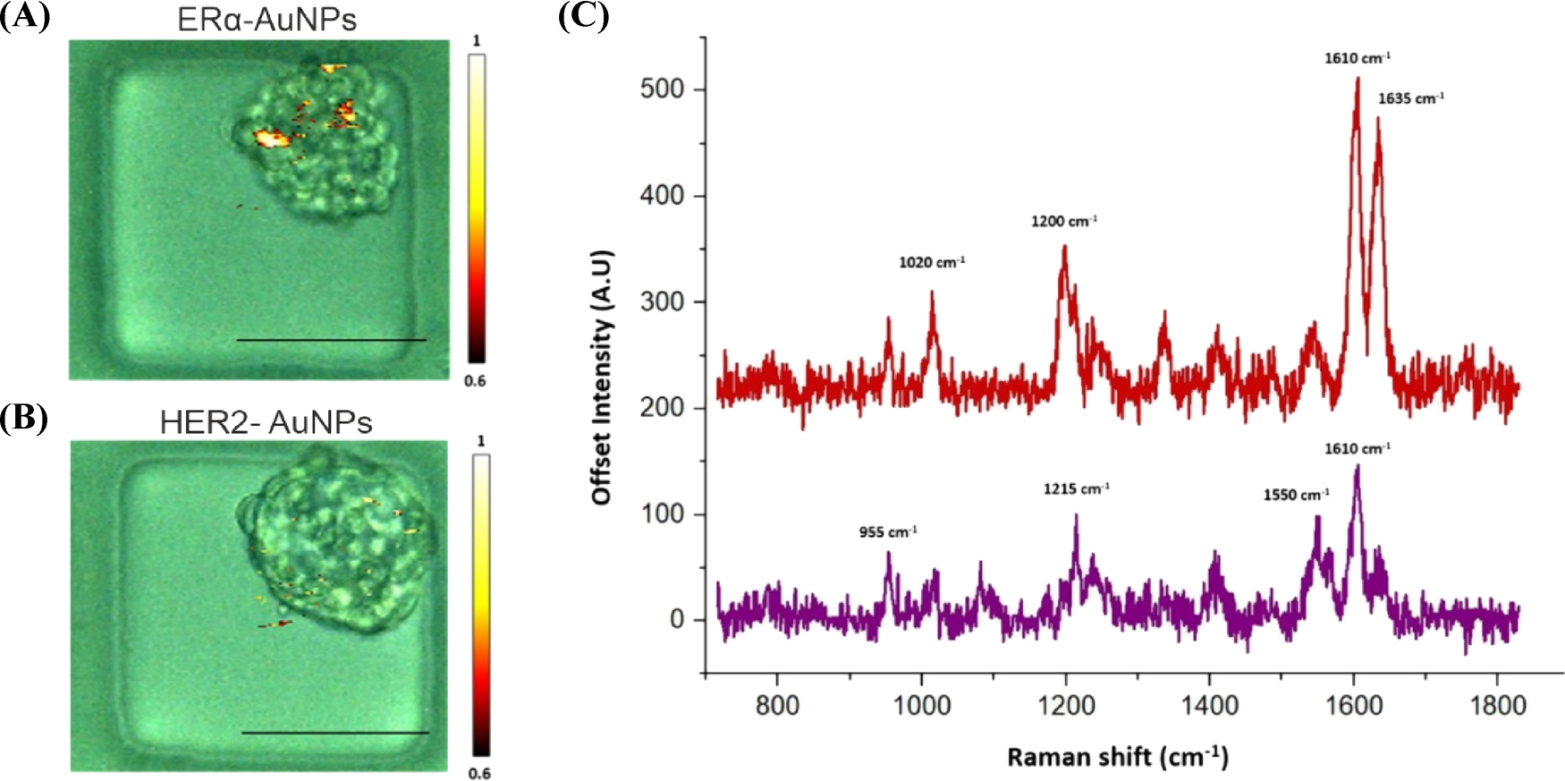
ERα-AuNP nanotags showed a strong targeting effect toward MCF-7 spheroids, while low HER2-AuNP accumulation was observed in MCF-7 spheroids due to nonspecific binding. (A) Bright-field image of an MCF-7 spheroid in a microfluidic channel merged with the corresponding SERS signal from ERα-AuNP nanotags. (B) Bright-field image of an MCF-7 spheroid in a microfluidic channel merged with the corresponding SERS signal from HER2-AuNP nanotags. (C) Representative SERS spectra from the highest signal points of ERα-AuNPs (red) and HER2-AuNPs (purple) in MCF-7 spheroids. The devices were turned upside down to facilitate interfacing with the Raman microscope, and the spheroids were then Raman-imaged using a laser excitation wavelength of 633 nm. 2D SERS mapping was carried out by focusing the laser of a Renishaw InVia Raman confocal microscope through the depth of the spheroids in the microwells in the microfluidic device. The minimum and maximum look up table (LUT) thresholds were set to exclude any poorly correlating or noisy spectra (min = 0.6, max = 1).

Prior to SERS analysis, the empty microfluidic devices were characterized to confirm that there was no overlapping signal between the devices and the SERS peaks from BPE and PPY Raman reporters (Figure S4). At day 4, ERα-AuNPs (60 pM, 2 h) or HER2-AuNPs (60 pM, 2 h) were injected into the microfluidic devices and incubated for 2 h before washing twice with PBS to remove any unbound nanotags. Previous work from our group has shown that 60 pM is an effective concentration to produce a high SERS response per cell without affecting the viability of MCF-7 cells.^50^ 2D SERS mapping from spheroids incubated with ERα-AuNPs demonstrated a high nanotag accumulation and a strong SERS signal, confirming the strong targeting effect of the ERα-AuNP nanotags to the MCF-7 spheroids (Figure 2A,C). In contrast, the spheroids treated with the nontargeted HER2-AuNP nanotags appeared to have a lower nanotag accumulation, demonstrated by the lower SERS signal corresponding to the PPY Raman reporter on HER2-AuNP nanotags (Figure 2B,C). These results confirmed that ERα-AuNP targeting was more effective in the spheroid tumors due to their overexpression of ERα. Since both the ERα-AuNP nanotags and the HER2-AuNP nanotags had the same SERS intensity, it is indicated that the lower HER2-AuNP accumulation in the MCF-7 spheroids was likely due to their low nonspecific binding in the spheroids.

**Figure 3:**
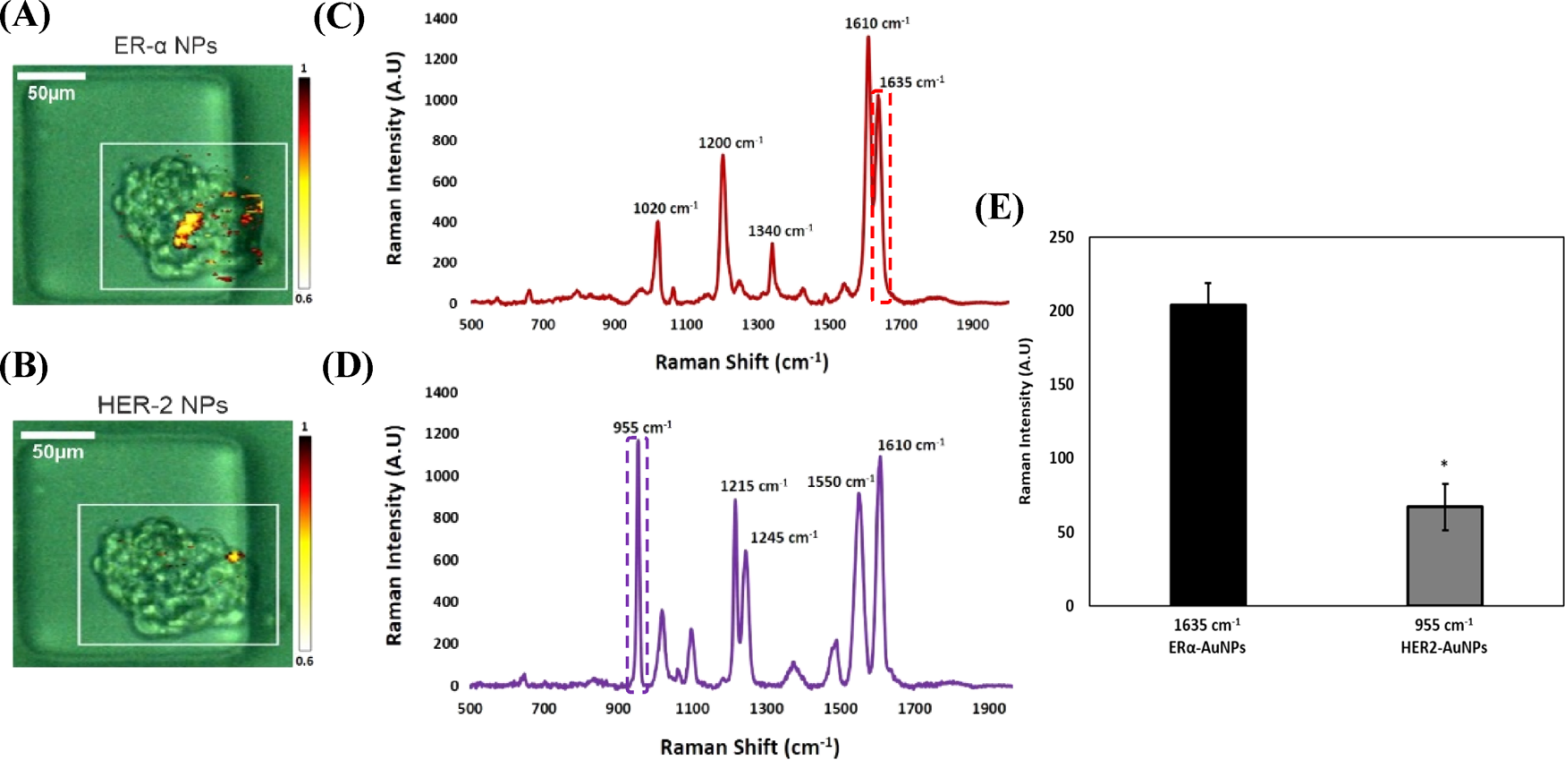
ERα-AuNPs showed a greater targeting effect and specificity for MCF-7 spheroids than HER2-AuNPs. MCF-7 spheroids incubated with the ERα + HER2-AuNP mixture (60 pM, 2 h) in microfluidic devices. The false color images correspond to the SERS signal from (A) ERα-AuNPs and (B) HER2-AuNPs within the same spheroid. The minimum and maximum look up table (LUT) thresholds were set to exclude any poorly correlating or noisy spectra (minimum = 0.6). (C) Reference spectra of ERα-AuNPs (BPE Raman reporter) (red) and (D) HER2-AuNPs (PPY Raman reporter) (purple) in H_2_O. The spectrum was collected using 100% laser power with 0.05 s accumulation time. The inset (dashed box) shows SERS intensity at 1635 cm^–1^ (red) that was selected as the representative peak for ERα-AuNPs (BPE Raman reporter) and SERS intensity at 955 cm^–1^ (red) that was selected as the representative peak for HER2-AuNPs (PPY Raman reporter). (E) Average Raman intensities at 1635 (ERα-AuNPs) and 955 cm^–1^ (HER2-AuNPs). The average of three samples from three independent biological replicates is shown. Error bars presented as mean ± S.D. * Significant difference (*p* < 0.05) in Student’s *t* test.

The multiplexing capability of SERS was also used to monitor the uptake of the two types of nanotags in the same analysis system under the same conditions and to confirm the specificity of ERα-AuNP nanotags for the MCF-7 spheroids. Hence, a 1:1 mixture of both the ERα-AuNP and HER2-AuNP nanotags were co-incubated with the spheroids. The results from the incubation of the targeted and nontargeted nanotag mixture in the spheroids confirmed that ERα-AuNP nanotags had a stronger targeting effect toward MCF-7 spheroids than HER2-AuNPs. Specifically, the ERα-AuNPs showed greater accumulation than HER2-AuNPs within the same spheroid (Figure 3A,B). Additionally, the spheroids had a significantly higher (2.7 times) Raman signal at 1635 cm^–1^, the representative peak of the BPE Raman reporter (Figure 3C) on ERα-AuNPs, than at 955 cm^–1^, the representative peak of the PPY Raman reporter (Figure 3D) on HER2-AuNPs (Figure 3E). Specifically, there was a 63% reduction in the PPY signal when the nanotags were attached to the spheroids, which was a 2.7-fold reduction from a 1:1 mixture. This result was calculated by taking into consideration the intensity of PPY in a 1:1 mixture (Figure S5A) and divided it by the fold reduction in signal from the spheroids (Figure S5B). The outcome from the calculation was then divided with the intensity of PPY from a 1:1 mixture and multiplied by 100 for the estimation of the percentage of the PPY intensity change in the spheroids.

The exact process that was followed for the quantification of PPY signal reduction is shown in Figure S5C. The quantification of the intensity ratio change confirmed that the targeted nanoparticles (ERα-AuNPs) accumulated more in the spheroids than the nontargeted nanoparticles (HER2-AuNPs) in the MCF-7 spheroids. In parallel, the ability of ERα-AuNPs to identify ERα-positive breast cancer spheroids using SERS was established. These results show promise for translating this approach to in vivo experiments where currently nontargeting nanotags^54,55^ are used.

### Targeting Effect of ERα-AuNP Nanotags in MCF-7 Spheroids: 3D SERS Imaging

3D SERS mapping was also used to investigate the uptake, surface adherence, and penetration abilities of AuNP nanotags into the MCF-7 spheroids and whether differences were present between targeted nanotags (ERα-AuNPs) and nontargeted ones (HER2-AuNPs). These differences could give an indication of whether the nanotags were uptaken by the spheroids or whether they just adhered to the surface of the spheroids. Therefore, 3D SERS mapping was carried out throughout the whole volume (with 200 μm diameter, 4188790.2 um^3^ volume in total) of MCF-7 spheroids treated with ERα-AuNPs (60 pM, 2 h) (Figure S6A) or HER2-AuNPs (60 pM, 2 h) (Figure S6B).

**Figure 4:**
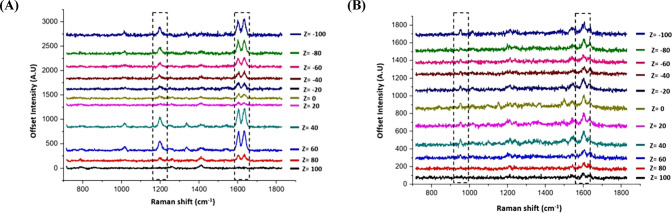
ERα-AuNP nanotags demonstrated greater accumulation within the MCF-7 spheroids than HER2-AuNPs. (A) Stacked 3D SERS spectra from ERα-AuNP nanotags generated at different *z* positions in the spheroid. (B) Stacked 3D SERS spectra from HER2-AuNPs generated at different *z* positions in the spheroid. Spheroids were mapped with a total volume of 200 μm^3^. *Z* = 0 represents the center of the spheroid.

The 3D SERS maps were collected using edge Streamline HR high confocality mode with a 3 μm step size resolution in the *x* and *y* directions and 4 μm between *z*-stacks. The representative average *z*-stacking results showed that there was a strong SERS signal from the ERα-AuNP nanotags obtained at depth within the spheroid volume indicating that the nanotags were targeting ERα (Figure 4A). MCF-7 spheroids incubated with HER2-AuNPs were also mapped using 3D SERS. The representative SERS *z*-stack signal from HER2-AuNPs showed that although the SERS signal was detected in the spheroids, it was much lower than that of ERα-AuNPs (Figure 4B). The increase of PPY and BPE signals suggests that there were a greater number of nanoparticles present at these locations within the cells, resulting in higher average SERS signals at these locations, which is consistent with the higher uptake of the ERα-AuNP nanoparticles by the spheroids. These 3D results confirmed the differences between the spheroids incubated with targeted ERα-AuNPs and the ones treated with HER2-AuNP nanotags. Additionally, the data indicate that ERα-AuNPs have greater retention into the spheroids than HER2-AuNP nanotags. However, future work comparing 3D SERS imaging in spheroids with other techniques, such as immunohistochemistry and transmission electron microscopy, should be conducted to investigate the penetration and retention capabilities of the nanotags. These preliminary studies indicate that HER2-AuNPs demonstrated much lower accumulation suggesting that their nontargeted uptake was nonspecific since there was no HER2 for them to target. The maximum SERS signal for each *z*-plane established the ability of the ERα-AuNPs to target ERα and implied the penetration of the nanotags in the breast cancer spheroids (Figure S7A,B). Therefore, microfluidics and SERS could potentially be utilized for the identification and characterization of breast cancer tumors ex vivo rapidly with sensitivity and specificity. The results reported here suggest that targeted nanoparticles result in higher nanoparticle uptake into spheroids and potentially tumors than nontargeted, nonspecific nanoparticle uptake. Therefore, this work shows how functionalized nanomaterials can be used in the future to characterize tumor areas by multiplexing the nanotags.

### Assessment of Fulvestrant Activity in MCF-7 Spheroids

**Figure 5:**
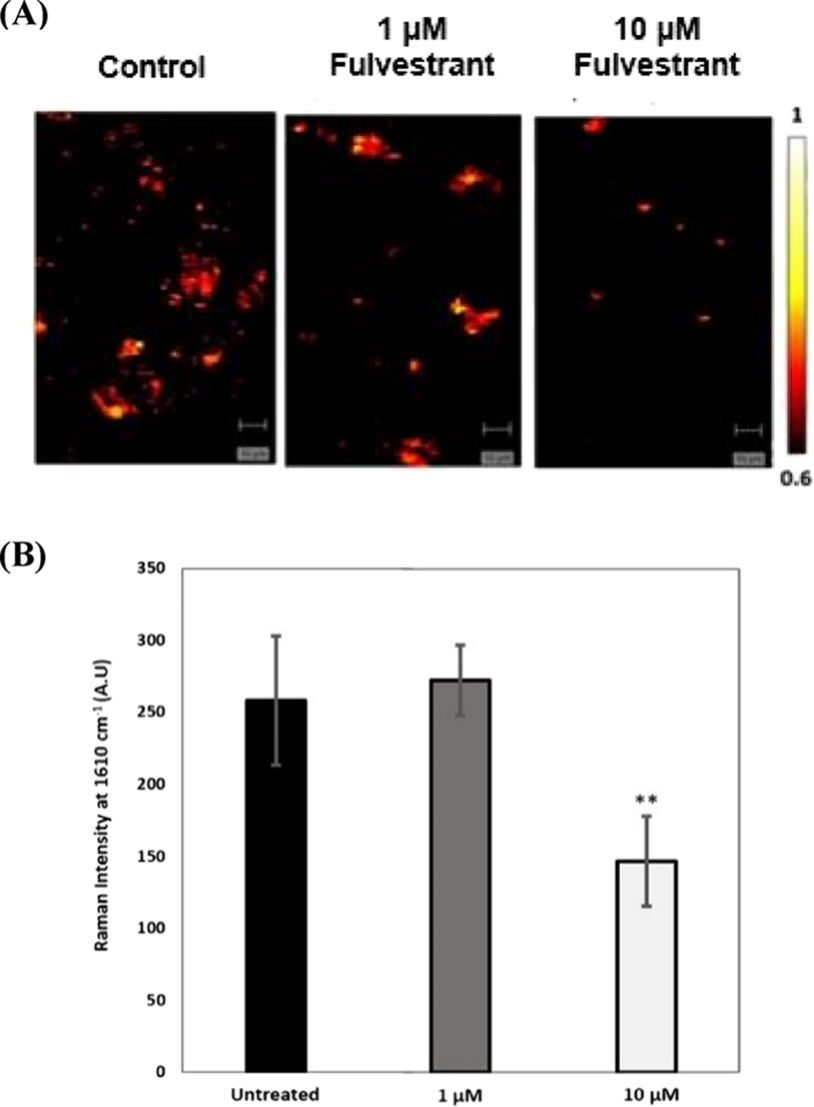
Treatment with 10 μM fulvestrant led to lower ERα-AuNP accumulation suggesting ERα reduction in MCF-7 spheroids. (A) SERS maps of untreated MCF-7 spheroids and spheroids treated with fulvestrant (1 and 10 μM) for 24 h and imaged usingERα-AuNP nanotags (60 pM, 2 h). The false colored images representing the ERα-AuNPs were generated using the Windows-based Raman Environment (WiRE - Renishaw plc) 4.4 software package on a Renishaw InVia microspectrometer and direct classical least squares analysis (DCLS) based on a BPE Raman reporter reference spectrum. The minimum and maximum look up table (LUT) thresholds were set to exclude any poorly correlating or noisy spectra (min = 0.6, max = 1). (B) Average Raman intensity at 1635 cm^–1^ (representative peak for the BPE Raman reporter on ERα-AuNPs). The average of three samples is shown. Error bars presented as mean ± S.D. * Significant difference (*p* < 0.05) in a one-way analysis of variance (ANOVA) test.

To further investigate the utility and benefits of the platform, the effect of nanoparticle uptake after drug treatment was investigated. Most patients with ERα-positive breast cancer benefit from endocrine therapy that targets the ERα pathway with higher efficacy and lower side effects.^56^ Endocrine therapy involves a class of drugs called selective estrogen receptor down-regulators (SERDs), which bind to ERα resulting in its degradation and downregulation.^57^ Fulvestrant is the first approved SERD for the treatment of ERα-positive breast cancer.^58,59^ Previous work from our lab has shown that MCF-7 cells treated with fulvestrant produced weaker SERS signals and lower accumulation of nanotags targeting the ERα receptor, indicating ERα degradation.^60^ Here, SERS and microfluidics were used to assess the efficacy of fulvestrant in spheroids. On day 3 of culture, fulvestrant (1 and 10 μM) was added to the spheroids for 24 h. Bright-field imaging was performed before and after fulvestrant addition to investigate any induced toxicity and structural differences in the spheroids (Figure S8A). The results showed that no significant difference in viability was produced on day 4 (Figure S8B,C). However, a significant reduction in the growth and increase in the disaggregation of the spheroids treated with 1 and 10 μM fulvestrant compared to the controls were observed on day 10 (Figure S8D). These results demonstrated that fulvestrant treatment increased the number of dead cells in the cancer spheroid model over time confirming its toxic effect. Therefore, day 4 was chosen as an optimal time point to assess the nanotag targeting effect based on the SERS response after 24 h of fulvestrant treatment. The spheroids were incubated on day 4 with ERα-AuNPs (60 pM, 2 h) and washed twice with PBS after 2 h to remove any unbound nanotags. SERS imaging was then carried out using a laser excitation wavelength of 633 nm (Figure S9). For SERS mapping, similarly sized spheroids were chosen from both the untreated and fulvestrant-treated samples. This step was carried out to increase the confidence that any eventual reduction of the SERS signal in the fulvestrant-treated spheroids was due to the ERα degradation and not due to spheroid size. SERS mapping showed that there was lower nanotag accumulation in the spheroids treated with 10 μM fulvestrant than with 1 μM fulvestrant and the untreated spheroids (Figure 5A).

Additionally, a statistically significant decrease (1.8 times) was observed in the SERS intensity at 1635 cm^–1^ (representative peak of the BPE Raman reporter attached to the ERα-AuNPs) after treatment with 10 μM of fulvestrant compared to the untreated spheroids (Figure 5B). These results confirmed that ERα degradation had occurred due to the fulvestrant treatment and validated that the targeting of ERα-AuNP nanotags toward ERα could be used to monitor the expression level in response to drug treatment. This is a significant first step in demonstrating the potential of using SERS and 3D cultures as a tool for preclinical drug validation. This has potential benefits in terms of the reduction in animal use for drug screening as only the most promising targets would be taken forward for in vivo experiments and ultimately increases positive outcomes and benefits patients through better informed treatment decision-making.

## Conclusions

Metal nanoparticles are commonly used in cancer as drug delivery,^61,62^ photothermal,^63,64^ and imaging agents.^65−67^ Here, we demonstrate that the multiplexing capabilities of SERS combined with targeted anti-ERα antibody-functionalized (ERα-AuNPs) and nontargeted anti-HER2 antibody-functionalized (HER2-AuNPs) nanotags can be successfully used to understand more about nanoparticle uptake in tumor spheroids with the advantages of high sensitivity and specificity. The results showed that SERS can provide a sensitive method for the analysis of 3D tumor models grown in microfluidic chips. Specifically, the combination of 3D spheroids and SERS was successfully applied to identify and classify live ERα-positive MCF-7 breast cancer spheroids and to compare the uptake of targeted and nontargeted nanoparticles into the 3D tumor model. The spheroids formed in the microfluidic device maintained their integrity and viability after SERS nanotag treatment, offering a versatile and robust methodology for scalable and multistep assays. A strong targeting effect of ERα-AuNPs was observed in MCF-7 spheroids compared to HER2-AuNPs. 3D SERS mapping revealed that the SERS signal was detected from areas within the inner part of the spheroid, suggesting the uptake and penetration of the nanotags into the spheroid. Furthermore, SERS allowed the assessment of fulvestrant activity on ERα expression levels post treatment. Specifically, the reduction of ERα protein after fulvestrant treatment was confirmed from the lower SERS signal in the fulvestrant-treated spheroids. Several studies have reported similar drug behaviors in 2D,^68−71^ which support our data in a 3D environment. This work highlights the importance of performing assays on 3D cultures that reflect better the tissue architecture and the cell-to-cell and cell-to-matrix interactions making them a great model to predict the drug responses in vivo. Future opportunities may involve multiplexed detection of biomarkers and investigation of the drug activity in patient-derived spheroids for extensive characterization of tumor tissue and its response to treatment.

## Supporting Information Available

Characterization of antibody–AuNP conjugates; Western blot for analysis of ERα and HER2 expressions in MCF-7 breast cancer cell lines; representative spectra of ERα-AuNP (BPE Raman reporter) and HER2-AuNP (PPY Raman reporter) mixtures in H_2_O; calculations performed to estimate the reduction of the PPY intensity in spheroids; 3D SERS map of an MCF-7 spheroid incubated with nanotags; 3D Raman mapping results and SERS spectra obtained at different *z*-axis points through MCF-7 spheroids with nanotags; bright-field and fluorescence imaging for spheroid treated with fulvestrant at different time points (PDF)

## Notes

All raw data pertaining to the manuscript can be accessed at https://doi.org/10.15129/c3d04bd3-64ef-4e7f-9d72-2cd1a3254a2f.

## References

[ref1] Ferlay J.; Soerjomataram I.; Dikshit R.; Eser S.; Mathers C.; Rebelo M.; Parkin D. M.; Forman D.; Bray F. Cancer incidence and mortality worldwide: sources, methods and major patterns in GLOBOCAN 2012. Int. J. Cancer 2015, 136, E359–E386. 10.1002/ijc.29210. 25220842

[ref2] Torre L. A.; Siegel R. L.; Ward E. M.; Jemal A. Global Cancer Incidence and Mortality Rates and Trends-An Update. Cancer Epidemiol., Biomarkers Prev. 2016, 25, 16–27. 10.1158/1055-9965.EPI-15-0578. 26667886

[ref3] UK, C. R. Breast Cancer Incidence (Invasive) Statistics: Breast Cancer Incidence (Invasive) Statistics: https://www.breastcancer.org/symptoms/understand_bc/statistics#:∼:text=About%201%20in%208%20U.S.,(in%20situ)%20breast%20cancer., Online Source. 2016.

[ref4] Korkaya H.; Liu S.; Wicha M. S. Breast cancer stem cells, cytokine networks, and the tumor microenvironment. J. Clin. Invest. 2011, 121, 3804–3809. 10.1172/JCI57099. 21965337 PMC3223613

[ref5] Quail D. F.; Joyce J. A. Microenvironmental regulation of tumor progression and metastasis. Nat. Med. 2013, 19, 1423–1437. 10.1038/nm.3394. 24202395 PMC3954707

[ref6] Mao Y.; Keller E. T.; Garfield D. H.; Shen K.; Wang J. Stromal cells in tumor microenvironment and breast cancer. Cancer Metastasis Rev. 2013, 32, 303–315. 10.1007/s10555-012-9415-3. 23114846 PMC4432936

[ref7] Jernström S.; Hongisto V.; Leivonen S. K.; Due E. U.; Tadele D. S.; Edgren H.; Kallioniemi O.; Perälä M.; Mælandsmo G. M.; Sahlberg K. K. Drug-screening and genomic analyses of HER2-positive breast cancer cell lines reveal predictors for treatment response. Breast Cancer: Targets Ther. 2017, Volume 9, 185–198. 10.2147/BCTT.S115600. PMC5367762 28356768

[ref8] Imamura Y.; Mukohara T.; Shimono Y.; Funakoshi Y.; Chayahara N.; Toyoda M.; Kiyota N.; Takao S.; Kono S.; Nakatsura T.; Minami H. Comparison of 2D- and 3D-culture models as drug-testing platforms in breast cancer. Oncol. Rep. 2015, 33, 1837–1843. 10.3892/or.2015.3767. 25634491

[ref9] Yamada K. M.; Cukierman E. Modeling tissue morphogenesis and cancer in 3D. Cell 2007, 130, 601–610. 10.1016/j.cell.2007.08.006. 17719539

[ref10] DelNero P.; Lane M.; Verbridge S. S.; Kwee B.; Kermani P.; Hempstead B.; Stroock A.; Fischbach C. 3D culture broadly regulates tumor cell hypoxia response and angiogenesis via pro-inflammatory pathways. Biomaterials 2015, 55, 110–118. 10.1016/j.biomaterials.2015.03.035. 25934456 PMC4417672

[ref11] Faute M. A. D.; Laurent L.; Ploton D.; Poupon M. F.; Jardillier J. C.; Bobichon H. Distinctive alterations of invasiveness, drug resistance and cell-cell organization in 3D-cultures of MCF-7, a human breast cancer cell line, and its multidrug resistant variant. Clin. Exp. Metastasis 2002, 19, 161–167. 10.1023/A:1014594825502. 11964080

[ref12] Fang Y.; Eglen R. M. Three-Dimensional Cell Cultures in Drug Discovery and Development. SLAS Discovery 2017, 22, 456–472. 10.1177/1087057117696795. 28520521 PMC5448717

[ref13] Breslin S.; O’Driscoll L. Three-dimensional cell culture: the missing link in drug discovery. Drug Discovery Today 2013, 18, 240–249. 10.1016/j.drudis.2012.10.003. 23073387

[ref14] Jeon J. S.; Bersini S.; Gilardi M.; Dubini G.; Charest J. L.; Moretti M.; Kamm R. D. Human 3D vascularized organotypic microfluidic assays to study breast cancer cell extravasation. Proc. Natl. Acad. Sci. 2015, 112, 214–219. 10.1073/pnas.1417115112. 25524628 PMC4291627

[ref15] Yang Y.; Yang X.; Zou J.; Jia C.; Hu Y.; Du H.; Wang H. Evaluation of photodynamic therapy efficiency using an in vitro three-dimensional microfluidic breast cancer tissue model. Lab Chip 2015, 15, 735–744. 10.1039/C4LC01065E. 25428803

[ref16] Sackmann E. K.; Fulton A. L.; Beebe D. J. The present and future role of microfluidics in biomedical research. Nature 2014, 507, 181–189. 10.1038/nature13118. 24622198

[ref17] Mehling M.; Tay S. Microfluidic cell culture. Curr. Opin. Biotechnol. 2014, 25, 95–102. 10.1016/j.copbio.2013.10.005. 24484886

[ref18] Du G.; Fang Q.; den Toonder J. M. J. Microfluidics for cell-based high throughput screening platforms - A review. Anal. Chim. Acta 2016, 903, 36–50. 10.1016/j.aca.2015.11.023. 26709297

[ref19] Salameh T. S.; Le T. T.; Nichols M. B.; Bauer E.; Cheng J.; Camarillo I. G. An ex vivo co-culture model system to evaluate stromal–epithelial interactions in breast cancer. Int. J. Cancer 2013, 132, 288–296. 10.1002/ijc.27672. 22696278

[ref20] MacKerron C.; Robertson G.; Zagnoni M.; Bushell T. J. A Microfluidic Platform for the Characterisation of CNS Active Compounds. Sci. Rep. 2017, 7, 15692 10.1038/s41598-017-15950-0. 29146949 PMC5691080

[ref21] Migliozzi D.; Nguyen H. T.; Gijs M. A. M. Combining fluorescence-based image segmentation and automated microfluidics for ultrafast cell-by-cell assessment of biomarkers for HER2 type breast carcinoma. J. Biomed. Opt. 2018, 24, 983–991. 10.1117/1.JBO.24.2.021204. PMC6987647 30484294

[ref22] Lin S. W.; Chang C. H.; Lin C. H. High-throughput Fluorescence Detections in Microfluidic Systems. Genomic Med., Biomarkers, Health Sci.. 2011, 3, 27–38. 10.1016/S2211-4254(11)60005-8.

[ref23] Willner M. R.; McMillan K. S.; Graham D.; Vikesland P. J.; Zagnoni M. Surface-Enhanced Raman Scattering Based Microfluidics for Single-Cell Analysis. Anal. Chem. 2018, 90, 12004–12010. 10.1021/acs.analchem.8b02636. 30230817

[ref24] Hussain A.; Pu H.; Sun D. W. Measurements of lycopene contents in fruit: A review of recent developments in conventional and novel techniques. Trends Food Sci. Technol. 2019, 59, 758–769. 10.1080/10408398.2018.1518896. 30582342

[ref25] Kamińska A.; Winkler K.; Kowalska A.; Witkowska E.; Szymborski T.; Janeczek A.; Waluk J. SERS-based Immunoassay in a Microfluidic System for the Multiplexed Recognition of Interleukins from Blood Plasma: Towards Picogram Detection. Sci. Rep. 2017, 7, 1 10.1038/s41598-017-11152-w. 28878312 PMC5587571

[ref26] Gao R.; Lv Z.; Mao Y.; Yu L.; Bi X.; Xu S.; Cui J.; Wu Y. SERS-Based Pump-Free Microfluidic Chip for Highly Sensitive Immunoassay of Prostate-Specific Antigen Biomarkers. ACS Sens. 2019, 4, 938–943. 10.1021/acssensors.9b00039. 30864786

[ref27] Aberasturi D. J.; Lacey M. H.; Litti L.; Langer J.; Liz-Marzán L. M. X.; Xu S.; Cui J.; Wu Y. Using SERS Tags to Image the Three-Dimensional Structure of Complex Cell Models. Adv. Funct. Mater. 2020, 30, 1909655 10.1002/adfm.201909655.

[ref28] Altunbek M.; Çetin D.; Suludere Z.; Çulha M. Surface-enhanced Raman spectroscopy based 3D spheroid culture for drug discovery studies. Talanta 2019, 191, 390–399. 10.1016/j.talanta.2018.08.087. 30262075

[ref29] Albrecht M. G.; Creighton J. A. Anomalously intense Raman spectra of pyridine at a silver electrode. J. Am. Chem. Soc. 1977, 99, 5215–5217. 10.1021/ja00457a071.

[ref30] Liao P. F.; Wokaun A. Lightning rod effect in surface enhanced Raman scattering. J. Chem. Phys. 1982, 76, 751 10.1063/1.442690.

[ref31] Asiala S. M.; Schultz Z. D. Characterization of hotspots in a highly enhancing SERS substrate. Analyst 2011, 136, 4472–4479. 10.1039/c1an15432j. 21946698 PMC3197236

[ref32] Faulds K.; McKenzie F.; Smith W. E.; Graham D. Quantitative simultaneous multianalyte detection of DNA by dual-wavelength surface-enhanced resonance Raman scattering. Am. Ethnol. 2007, 46, 1829–1831. 10.1002/anie.200604265. 17262874

[ref33] Gracie K.; Correa E.; Mabbott S.; Dougan J. A.; Graham D.; Goodacre R.; Faulds K. Simultaneous detection and quantification of three bacterial meningitis pathogens by SERS. Chem. Sci. 2014, 5, 1030–1040. 10.1039/C3SC52875H.

[ref34] Lee S.; Chon H.; Lee J.; Ko J.; Chung B. H.; Lim D. W.; Choo J. Rapid and sensitive phenotypic marker detection on breast cancer cells using surface-enhanced Raman scattering (SERS) imaging. Biosens. Bioelectron. 2014, 51, 238–243. 10.1016/j.bios.2013.07.063. 23973735

[ref35] Davis R.; Campbell J.; Burkitt S.; Qiu Z.; Kang S.; Mehraein M.; Miyasato D.; Salinas H.; Liu J.; Zavaleta C. A Raman Imaging Approach Using CD47 Antibody-Labeled SERS Nanoparticles for Identifying Breast Cancer and Its Potential to Guide Surgical Resection. Nanomaterials 2018, 8, 953 10.3390/nano8110953. PMC6265869 30463284

[ref36] Harmsen S.; Wall M. A.; Huang R.; Kircher M. F. Cancer imaging using surface-enhanced resonance Raman scattering nanoparticles. Nat. Protoc. 2017, 12, 1400–1414. 10.1038/nprot.2017.031. 28686581 PMC5694346

[ref37] El-Said W. A.; Yoon J.; Choi J. W. Nanostructured surfaces for analysis of anticancer drug and cell diagnosis based on electrochemical and SERS tools. Nano Convergence. 2018, 5, 1 10.1186/s40580-018-0143-4. 29721403 PMC5913382

[ref38] Panikar S. S.; Ramírez-García G.; Sidhik S.; Lopez-Luke T.; Rodriguez-Gonzalez C.; Ciapara I. H.; Castillo P. S.; Camacho-Villegas T.; De La Rosa E. Ultrasensitive SERS Substrate for Label-Free Therapeutic-Drug Monitoring of Paclitaxel and Cyclophosphamide in Blood Serum. Anal. Chem. 2019, 91, 2100–2111. 10.1021/acs.analchem.8b04523. 30580508

[ref39] Ji M.; Lewis S.; Camelo-Piragua S.; Ramkissoon S. H.; Snuderl M.; Venneti S.; Fisher-Hubbard A.; Garrard M.; Fu D.; Wang A. C.; Heth J. A.; Maher C. O.; Sanai N.; Johnson T. D.; Freudiger C. W.; Sagher O.; Xie X. S.; Orringer D. A. Detection of human brain tumor infiltration with quantitative stimulated Raman scattering microscopy. Sci. Transl. Med. 2015, 7, 309ra163–309ra163. 10.1126/scitranslmed.aab0195. PMC4900155 26468325

[ref40] Smith B. R.; Gambhir S. S. Nanomaterials for In Vivo Imaging. Chem. Rev. 2017, 117, 901–986. 10.1021/acs.chemrev.6b00073. 28045253

[ref41] Hashizume H.; Baluk P.; Morikawa S.; McLean J. W.; Thurston G.; Roberge S.; Jain R. K.; McDonald D. M. Openings between defective endothelial cells explain tumor vessel leakiness. Am. J. Pathol. 2000, 156, 1363–1380. 10.1016/S0002-9440(10)65006-7. 10751361 PMC1876882

[ref42] Smith B. R.; Zavaleta C.; Rosenberg J.; Tong R.; Ramunas J.; Liu Z.; Dai H.; Gambhir S. S. High-resolution, serial intravital microscopic imaging of nanoparticle delivery and targeting in a small animal tumor model. Nano Today 2013, 8, 126 10.1016/j.nantod.2013.02.004. PMC3836612 24273594

[ref43] Chen W.; Cormode D. P.; Fayad Z. A.; Mulder W. J. M. Nanoparticles as magnetic resonance imaging contrast agents for vascular and cardiac diseases. Wiley Interdisciplinary Reviews: Nanomedicine and Nanobiotechnology. 2011, 3, 146–161. 10.1002/wnan.114. 20967875 PMC3256288

[ref44] Bertrand N.; Wu J.; Xu X.; Kamaly N.; Farokhzad O. C. Cancer nanotechnology: the impact of passive and active targeting in the era of modern cancer biology. Adv. Drug Delivery Rev. 2014, 66, 2–25. 10.1016/j.addr.2013.11.009. PMC4219254 24270007

[ref45] Shi J.; Xiao Z.; Kamaly N.; Farokhzad O. C. Self-Assembled Targeted Nanoparticles: Evolution of Technologies and Bench to Bedside Translation. Acc. Chem. Res. 2011, 44, 1123–1134. 10.1021/ar200054n. 21692448

[ref46] Kirpotin D. B.; Drummond D. C.; Shao Y.; Shalaby M. R.; Hong K.; Nielsen U. B.; Marks J. D.; Benz C. C.; Park J. W. Antibody targeting of long-circulating lipidic nanoparticles does not increase tumor localization but does increase internalization in animal models. Cancer Res. 2006, 66, 6732–6740. 10.1158/0008-5472.CAN-05-4199. 16818648

[ref47] De La Zerda A.; Zavaleta C.; Keren S.; Vaithilingam S.; Bodapati S.; Liu Z.; Levi J.; Smith B. R.; Ma T.-J.; Oralkan O.; Cheng Z.; Chen X.; Dai H.; Khuri-Yakub B. T.; Gambhir S. S. Carbon nanotubes as photoacoustic molecular imaging agents in living mice. Nat. Nanotechnol. 2008, 3, 557–562. 10.1038/nnano.2008.231. 18772918 PMC2562547

[ref48] Mulholland T.; McAllister M.; Patek S.; Flint D.; Underwood M.; Sim A.; Edwards J.; Zagnoni M. Drug screening of biopsy-derived spheroids using a self-generated microfluidic concentration gradient. Sci. Rep. 2018, 8, 14672 10.1038/s41598-018-33055-0. 30279484 PMC6168499

[ref49] Turkevich J.; Stevenson P. C.; Hillier J. A study of the nucleation and growth processes in the synthesis of colloidal gold. Faraday Discuss. 1951, 11, 55 10.1039/DF9511100055.

[ref50] Kapara A.; Brunton V.; Graham D.; Faulds K. Investigation of cellular uptake mechanism of functionalised gold nanoparticles into breast cancer using SERS. Chem. Sci. 2020, 11, 5819–5829. 10.1039/D0SC01255F. PMC8159335 34094083

[ref51] Bartczak D.; Kanaras A. G. Preparation of Peptide-Functionalized Gold Nanoparticles Using One Pot EDC/Sulfo-NHS Coupling. Langmuir 2011, 27, 10119–10123. 10.1021/la2022177. 21728291

[ref52] Piruska A.; Nikcevic I.; Lee S. H.; Ahn C.; Heineman W. R.; Limbach P. A.; Seliskar C. J. The autofluorescence of plastic materials and chips measured under laser irradiation. Lab Chip 2005, 5, 1348–1354. 10.1039/b508288a. 16286964

[ref53] Mata A.; Fleischman A. J.; Roy S. Characterization of Polydimethylsiloxane (PDMS) Properties for Biomedical Micro/Nanosystems. Biomed. Microdevices 2005, 7, 281–293. 10.1007/s10544-005-6070-2. 16404506

[ref54] Nicolson F.; Andreiuk B.; Andreou C.; Hsu H. T.; Rudder S.; Kircher M. F. Non-invasive In Vivo Imaging of Cancer Using Surface-Enhanced Spatially Offset Raman Spectroscopy (SESORS). Theranostics. 2019, 9, 5899–5913. 10.7150/thno.36321. 31534527 PMC6735365

[ref55] Bailly A. L.; Correard F.; Popov A.; Tselikov G.; Chaspoul F.; Appay R.; Al-Kattan A.; Kabashin A. V.; Braguer D.; Esteve M. A. In vivo evaluation of safety, biodistribution and pharmacokinetics of laser-synthesized gold nanoparticles. Sci. Rep. 2019, 9, 1 10.1038/s41598-019-48748-3. 31501470 PMC6734012

[ref56] Bonotto M.; Gerratana L.; Di Maio M.; De Angelis C.; Cinausero M.; Moroso S.; Milano M.; Stanzione B.; Gargiulo P.; Iacono D.; Minisini A. M.; Mansutti M.; Fasola G.; de Placido S.; Arpino G.; Puglisi F. Chemotherapy versus endocrine therapy as first-line treatment in patients with luminal-like HER2-negative metastatic breast cancer: A propensity score analysis. Breast. 2017, 31, 114–120. 10.1016/j.breast.2016.10.021. 27837704

[ref57] Patel H. K.; Bihani T. Selective estrogen receptor modulators (SERMs) and selective estrogen receptor degraders (SERDs) in cancer treatment. Pharmacol. Ther. 2018, 186, 1–24. 10.1016/j.pharmthera.2017.12.012. 29289555

[ref58] Croxtall J. D.; McKeage K. Fulvestrant: a review of its use in the management of hormone receptor-positive metastatic breast cancer in postmenopausal women. Drugs 2011, 71, 363–380. 10.2165/11204810-000000000-00000. 21319872

[ref59] Nathan M. R.; Schmid P. A Review of Fulvestrant in Breast Cancer. Oncol. Ther. 2017, 5, 17–29. 10.1007/s40487-017-0046-2. 28680952 PMC5488136

[ref60] Kapara A.; Brunton V. G.; Graham D.; Faulds K. Characterisation of estrogen receptor alpha (ERα) expression in breast cancer cells and effect of drug treatment using targeted nanoparticles and SERS. Analyst 2020, 145, 7225–7233. 10.1039/D0AN01532F. 33164013

[ref61] Rizvi S. A. A.; Saleh A. M. Applications of nanoparticle systems in drug delivery technology. Saudi Pharm. J. 2018, 26, 64–70. 10.1016/j.jsps.2017.10.012. 29379334 PMC5783816

[ref62] McAughtrie S.; Faulds K.; Graham D. Surface enhanced Raman spectroscopy (SERS): potential applications for disease detection and treatment. Photochem. Rev. 2014, 21, 40–53. 10.1016/j.jphotochemrev.2014.09.002.

[ref63] Yang Z.; Sun Z.; Ren Y.; Chen X.; Zhang W.; Zhu X.; Mao Z.; Shen J.; Nie S. Advances in nanomaterials for use in photothermal and photodynamic therapeutics Reviews. Mol. Med. Rep. 2019, 20, 5–15. 10.3892/mmr.2019.10218. 31115497 PMC6579972

[ref64] Vines J. B.; Yoon J. H.; Ryu N. E.; Lim D. J.; Park H. Gold Nanoparticles for Photothermal Cancer Therapy. Front. Chem. 2019, 7, 398 10.3389/fchem.2019.00167. 31024882 PMC6460051

[ref65] Nune S. K.; Gunda P.; Thallapally P. K.; Lin Y. Y.; Laird Forrest M.; Berkland C. J. Nanoparticles for biomedical imaging. Expert Opin. Drug Delivery. 2009, 6, 1175–1194. 10.1517/17425240903229031. PMC3097035 19743894

[ref66] Chapman S.; Dobrovolskaia M.; Farahani K.; Goodwin A.; Joshi A.; Lee H.; Meade T.; Pomper M.; Ptak K.; Rao J.; Singh R.; Sridhar S.; Stern S.; Wang A.; Weaver J. B.; Woloschak G.; Yang L. Nanoparticles for cancer imaging: The good, the bad, and the promise. Nano Today 2013, 8, 454–460. 10.1016/j.nantod.2013.06.001. 25419228 PMC4240321

[ref67] Noonan J.; Asiala S. M.; Grassia G.; MacRitchie N.; Gracie K.; Carson J.; Moores M.; Girolami M.; Bradshaw A. C.; Guzik T. J.; Meehan G. R.; Scales H. E.; Brewer J. M.; McInnes I. B.; Sattar N.; Faulds K.; Garside P.; Meehan G. R. In vivo multiplex molecular imaging of vascular inflammation using surface-enhanced Raman spectroscopy. Theranostics. 2018, 8, 6195–6209. 10.7150/thno.28665. 30613292 PMC6299693

[ref68] Pickl M.; Ries C. H. Comparison of 3D and 2D tumor models reveals enhanced HER2 activation in 3D associated with an increased response to trastuzumab. Oncogene 2009, 28, 461–468. 10.1038/onc.2008.394. 18978815

[ref69] Lee G. Y.; Kenny P. A.; Lee E. H.; Bissell M. J. Three-dimensional culture models of normal and malignant breast epithelial cells. Nat. Methods 2007, 4, 359–365. 10.1038/nmeth1015. 17396127 PMC2933182

[ref70] Shaw K. R. M.; Wrobel C. N.; Brugge J. S. Use of three-dimensional basement membrane cultures to model oncogene-induced changes in mammary epithelial morphogenesis. J. Mammary Gland Biol. Neoplasia 2004, 9, 297–310. 10.1007/s10911-004-1402-z. 15838601 PMC1509102

[ref71] Gao Y.; Majumdar D.; Jovanovic B.; Shaifer C.; Lin P. C.; Zijlstra A.; Webb D. J.; Li D. A versatile valve-enabled microfluidic cell co-culture platform and demonstration of its applications to neurobiology and cancer biology. Biomed. Microdevices 2011, 13, 539–548. 10.1007/s10544-011-9523-9. 21424383 PMC3085600

