# Supplementary material for: Detection of Estrogen Receptor Alpha and Assessment of Fulvestrant Activity in MCF-7 Tumor Spheroids Using Microfluidics and SERS
Source: Anal Chem. 2021 Apr 2;93(14):5862–71. doi: 10.1021/acs.analchem.1c00188 (PMC8153394; doi:10.1021/acs.analchem.1c00188)
Supplement: Supplementary file 1 — ac1c00188_si_001.pdf [file ac1c00188_si_001.pdf]

Supporting Information

Detection of Estrogen Receptor Alpha and Assessment of Fulvestrant Activity in MCF-7 Tumour Spheroids Using Microfluidics and SERS

Anastasia Kapara[†,‡], Karla A. Findlay Paterson[§], Valerie Brunton[‡], Duncan Graham[†], Michele Zagnoni[§], Karen Faulds[†*]

[†] Centre for Molecular Nanometrology, Department of Pure and Applied Chemistry, Technology and Innovation Centre, University of Strathclyde, 99 George Street, Glasgow G1 1RD, UK

[‡] MRC Institute of Genetics and Molecular Medicine, Edinburgh Cancer Research UK Centre, University of Edinburgh, Western General Hospital, Crewe Road South, Edinburgh, EH4 2XU, UK

[§] Centre for Microsystems and Photonics, Department of Electronic and Electrical Engineering, Technology and Innovation Centre, University of Strathclyde, 99 George Street, Glasgow G1 1RD, UK

[*]Corresponding author: karen.faulds@strath.ac.uk

Contents

SI Figure S1 3
SI Figure S2 3
SI Figure S3 4
SI Figure S4 4
SI Figure S5 6
SI Figure S6 6
SI Figure S7 7
SI Figure S8 8
SI Figure S9 8

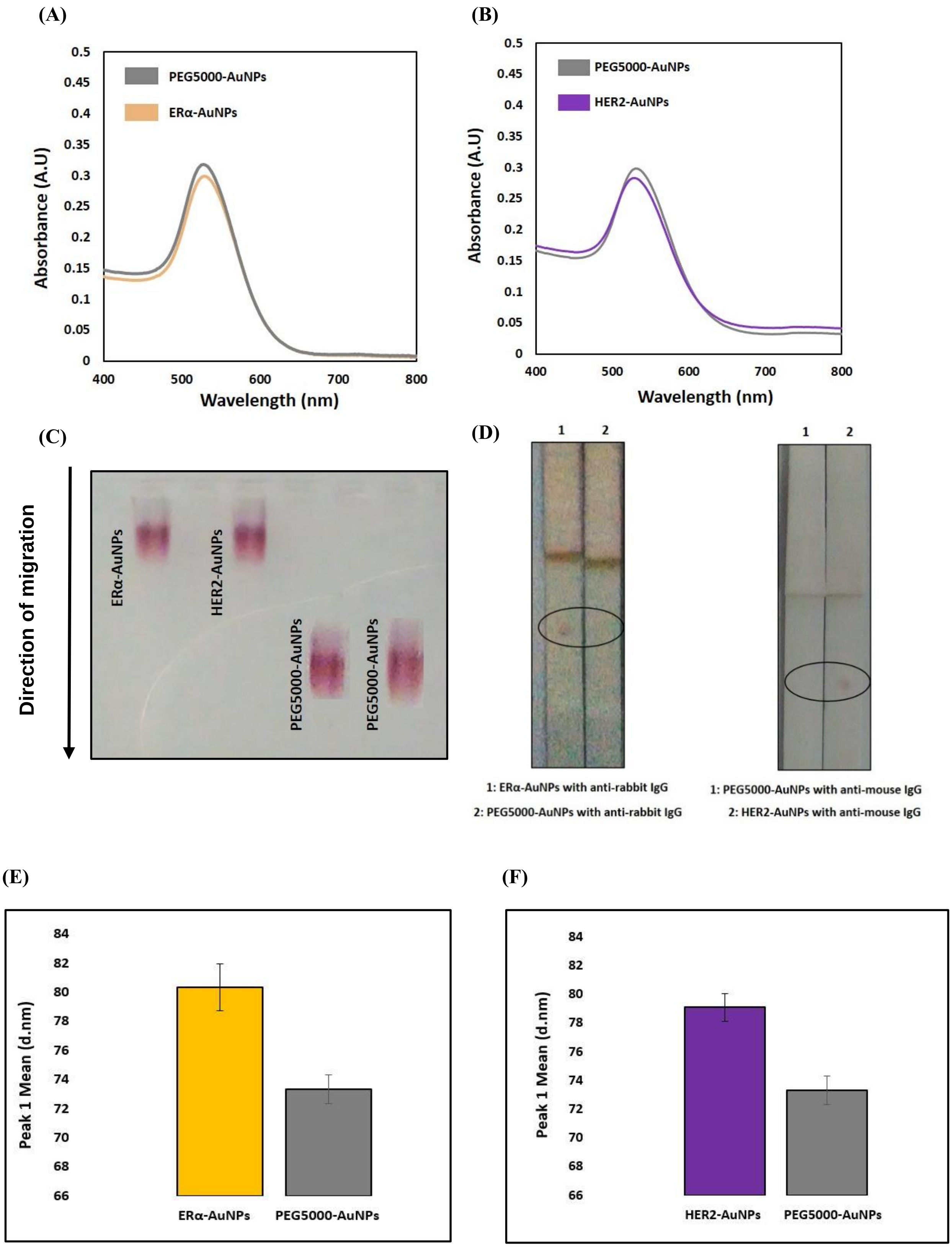
(A)
PEG5000-AuNPs
ERα-AuNPs
Absorbance (A.U)
Wavelength (nm)
(B)
PEG5000-AuNPs
HER2-AuNPs
Absorbance (A.U)
Wavelength (nm)
(C)
Direction of migration
ERα-AuNPs
HER2-AuNPs
PEG5000-AuNPs
PEG5000-AuNPs
(D)
1: ERα-AuNPs with anti-rabbit IgG
2: PEG5000-AuNPs with anti-rabbit IgG
1: PEG5000-AuNPs with anti-mouse IgG
2: HER2-AuNPs with anti-mouse IgG
(E)
Peak 1 Mean (d.nm)
ERα-AuNPs
PEG5000-AuNPs
(F)
Peak 1 Mean (d.nm)
HER2-AuNPs
PEG5000-AuNPs

SI Figure S1: (A) Extinction spectra of PEG5000-AuNPs (grey) and ERα-AuNPs (orange) nanotags. (B) Extinction spectra of PEG5000-AuNPs (grey) and HER2-AuNPs (purple) nanotags. (C) Agarose gel after electrophoresis showing the direction of migration and the distance travelled by PEG5000-AuNPs, ERα-AuNPs and HER2-AuNP nanotags. Gel electrophoresis is a method of separation and analysis, based on the size and charge of the samples being analysed. Samples travel through a gel matrix due to an electric charge being applied through the gel. Larger samples travel slower through the gel compared to smaller samples, which can travel faster through the porous matrix. (D) Lateral flow immunosorbent assay strips showing the spot from ERα-AuNPs and HER2-AuNPs on the detection zone of the nitrocellulose strip. The spot was present only in samples with the matching secondary IgG antibody for ERα (anti-rabbit) or HER2 (anti-mouse) applied. There was no detected spot when PEG5000-AuNPs was tested with the anti- rabbit IgG and anti-mouse IgG confirming the successful binding of the anti-ERα antibody and anti-HER2 to the AuNPs surface. (E-F) Differential light scanning analysis (DLS) of PEG5000-AuNPs (grey), ERα-AuNPs (orange) and HER2AuNPs nanotags confirmed the successful functionalisation of the anti-ERα antibody and the anti-HER2 antibody since the hydrodynamic diameter of AuNPs increased as each layer was added. ERα-AuNP nanotags were 80.3±1.6 d.nm and the HER2-AuNPs were 79.8±0.96 d.nm while the PEG5000-AuNPs were 73.0±1.0 d.nm. The HS-PEG5000-COOH was used for the functionalisation to prevent nonspecific interactions between the functionalised nanotags and other cellular components. Additionally, it provides the functional group for the covalent the attachment of the anti ERα antibody or anti-HER2 antibody. The HS-PEG5000-COOH was able to bind to the gold surface due to the strong binding affinity of the thiol group to gold surface while BPE and PPY molecules bind to the AuNPs via the nitrogen (N) atom of the pyridyl ring .

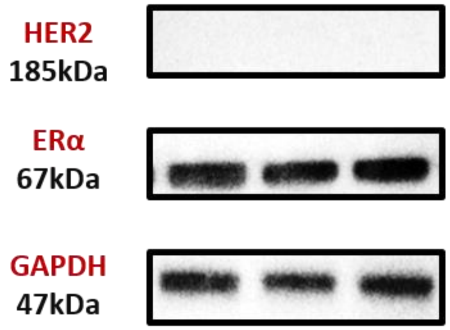

SI Figure S2: ERα and HER2 expression in MCF-7 breast cancer cell lines. Cell lysates were prepared from MCF-7 cells and western blot analysis carried out using an antibody to ERα and an antibody to HER2. GAPDH was used as a loading control. MCF-7 cells showed high ERα expression (at 67 kDa) and no detectable expression of HER2 (expected at 185 kDa). The experiment performed in three different biological samples.

(A)

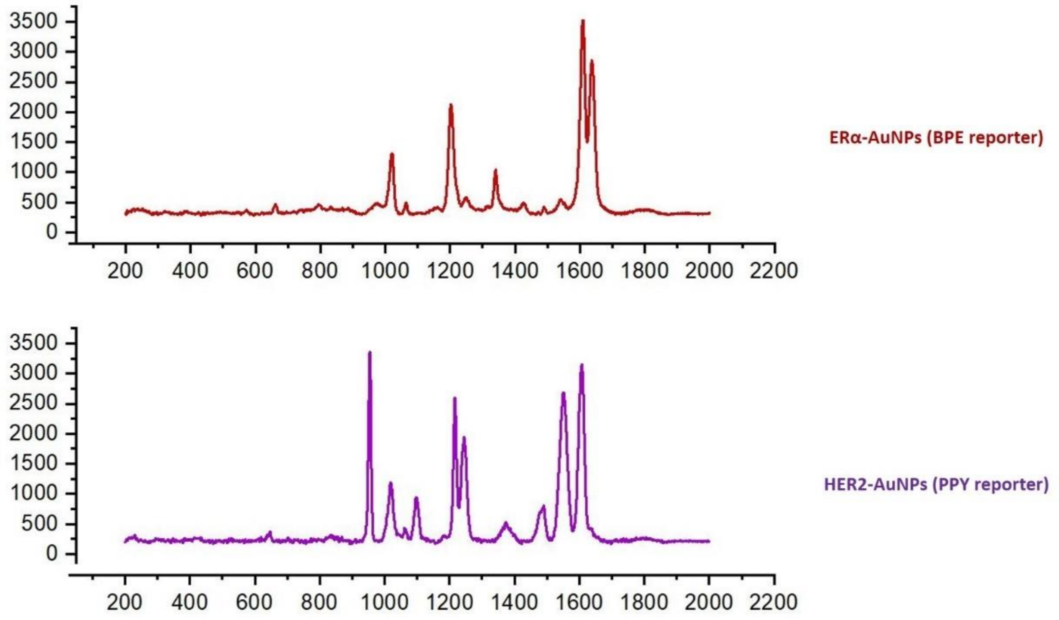

(B)

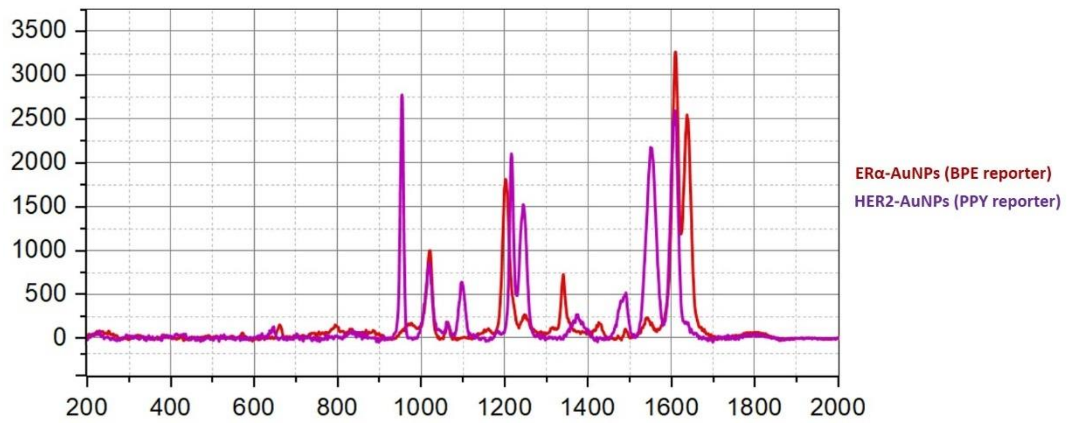

SI Figure S3: (A) Representative spectra ERα-AuNPs (BPE Raman reporter) and HER2-AuNPs (PPY Raman reporter) mixture in H20. Both nanotags were synthesised to have the same signal intensity per number of NPs to the allow comparison in SERS intensity. (B) SERS spectra of ERα-AuNPs (BPE Raman reporter) and HER2-AuNPs (PPY Raman reporter) mixture in H_20. All spectra were collected using 100% laser power with 1 s accumulation time. The inset (dashed box) shows SERS intensity at 1635 cm^{-1} (red) that was selected as representative peaks for ERα-AuNPs (BPE Raman reporter) and SERS intensity at 955 cm^{-1} (red) that was selected as representative peaks HER2-AuNPs (PPY Raman reporter).

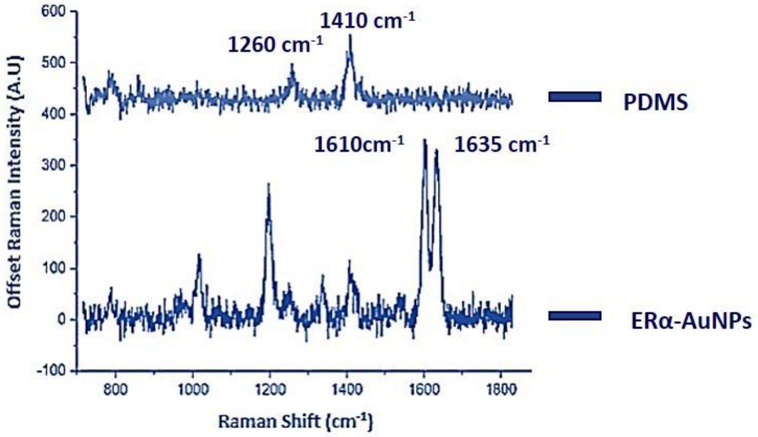

SI Figure S4: Prior to SERS analysis, the empty microfluidic devices were characterised to identify any unwanted background Raman signal. Although PDMS gave a low intensity Raman spectrum, the peaks at 1260 cm-1 and 1410 cm-1 did not overlap with the SERS peaks from BPE and PPY Raman reporters on the nanotags. The peak from PDMS at 1410 cm-1 does not overlap with the Raman peak at 1610 cm-1 (representative peak for ERα-AuNP nanotags). Therefore, PDMS does not interfere with the SERS spheroids mapping results. SERS spectra from PDMS were taken from an empty microfluidic device.

(A)

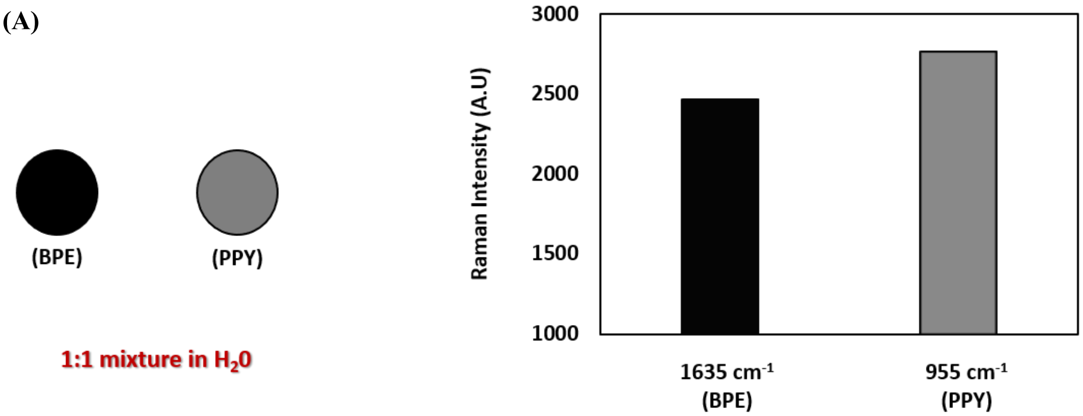

BPE Raman Intensity: 2460 A.U

PPY Raman Intensity: 2763 A.U

Raman Intensity Ratio (BPE/PPY): 0.89

(B)

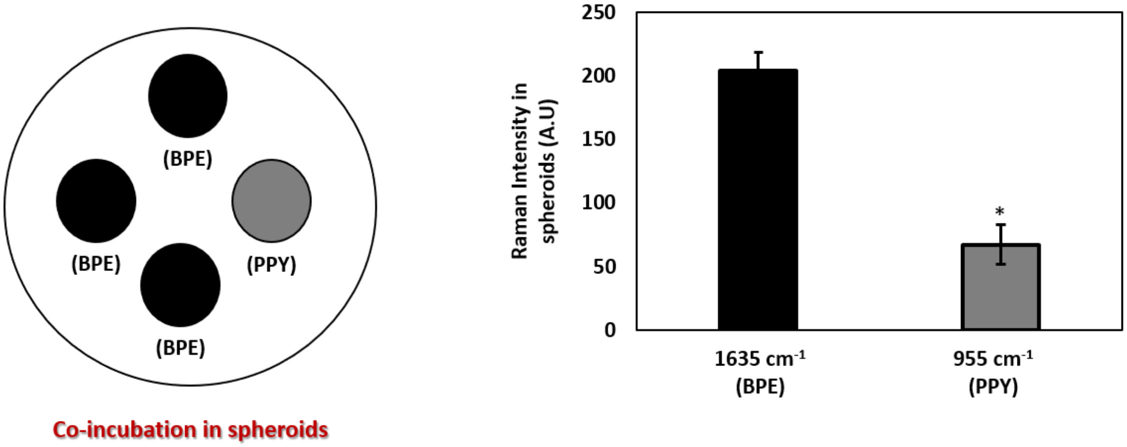

BPE Raman Intensity in spheroids: 204 A.U

PPY Raman Intensity in spheroids: 75 A.U

Raman Intensity Ratio in Spheroids (BPE/PPY): 2.7

(C)

1) Change in PPY intensity from 1:1 mixture (SI, Figure S5A) to incubation in spheroids (SI, Figure S5B) would be:
2763 / 2.7 = 1023

2) Hence, the percentage of PPY intensity change would be:
1023 / 2763 *100 = 37%

3) Therefore, the reduction of PPY in spheroids would be:
100 – 37 = 63%

SI Figure S5: SI Figure S5: (A) Raman intensity at 1635 cm^{-1} (ERα-AuNPs, BPE reporter) and 955 cm^{-1} (HER2-AuNPs, PPY reporter) of 1:1 mixture of nanotags in H_20. (B) Average Raman intensity at 1635 cm^{-1} (ERα-AuNPs, BPE reporter) and 955 cm^{-1} (HER2-AuNPs, PPY reporter) co-incubated in MCF-7 spheroids. The black and grey circles on both (A) and (B) are a visual representation of the ratio of ERα-AuNPs and HER2-AuNPs. (C) Calculations performed to estimate the reduction of PPY intensity in spheroids. (1) The intensity of PPY in H20 (2763 A.U) was divided with the ratio of BPE:PPY in spheroids (2.7) to estimate the change in PPY intensity in the spheroids (1023). (2) The outcome from calculation (1) was then divided with the intensity of PPY in H20 (2763 A.U) and multiplied by 100 for the estimation of the percentage of PPY intensity change in the spheroids. (3) The percentage was then subtracted from 100 and it was calculated that there was a 63% reduction in the PPY signal when attaching to the spheroid.

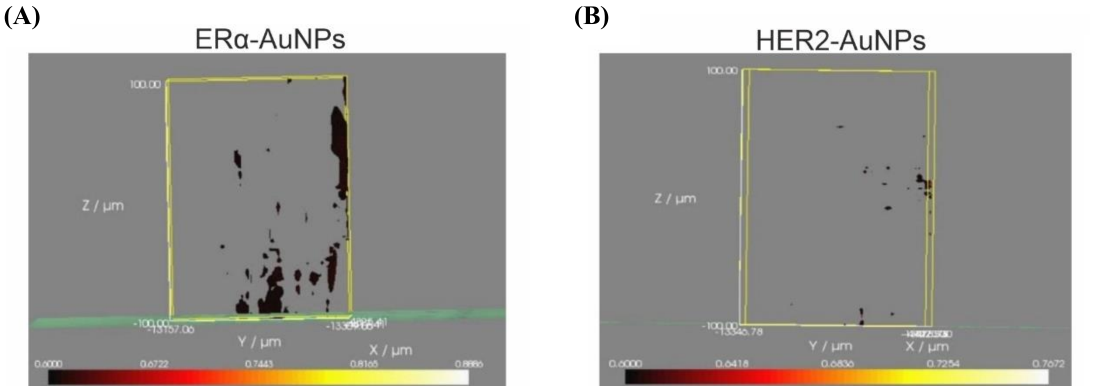

SI Figure S6: SI Figure S6: ERα-AuNP nanotags demonstrated greater accumulation within the MCF-7 spheroids in comparison to HER2-AuNPs. (A) 3D SERS map of an MCF-7 spheroid incubated with ERα-AuNP nanotags (60 pM, 2 h) in the microfluidic device. (B) 3D SERS map of an MCF-7 spheroid incubated with HER2-AuNPs (60 pM, 2 h) in the microfluidic device. Spheroids were mapped with a total volume of 200 µm3. False colour represents the areas where SERS signal from nanotags was present throughout the spheroids. The minimum look up table (LUT) threshold was set to exclude any poorly correlating or noisy spectra (min= 0.6).

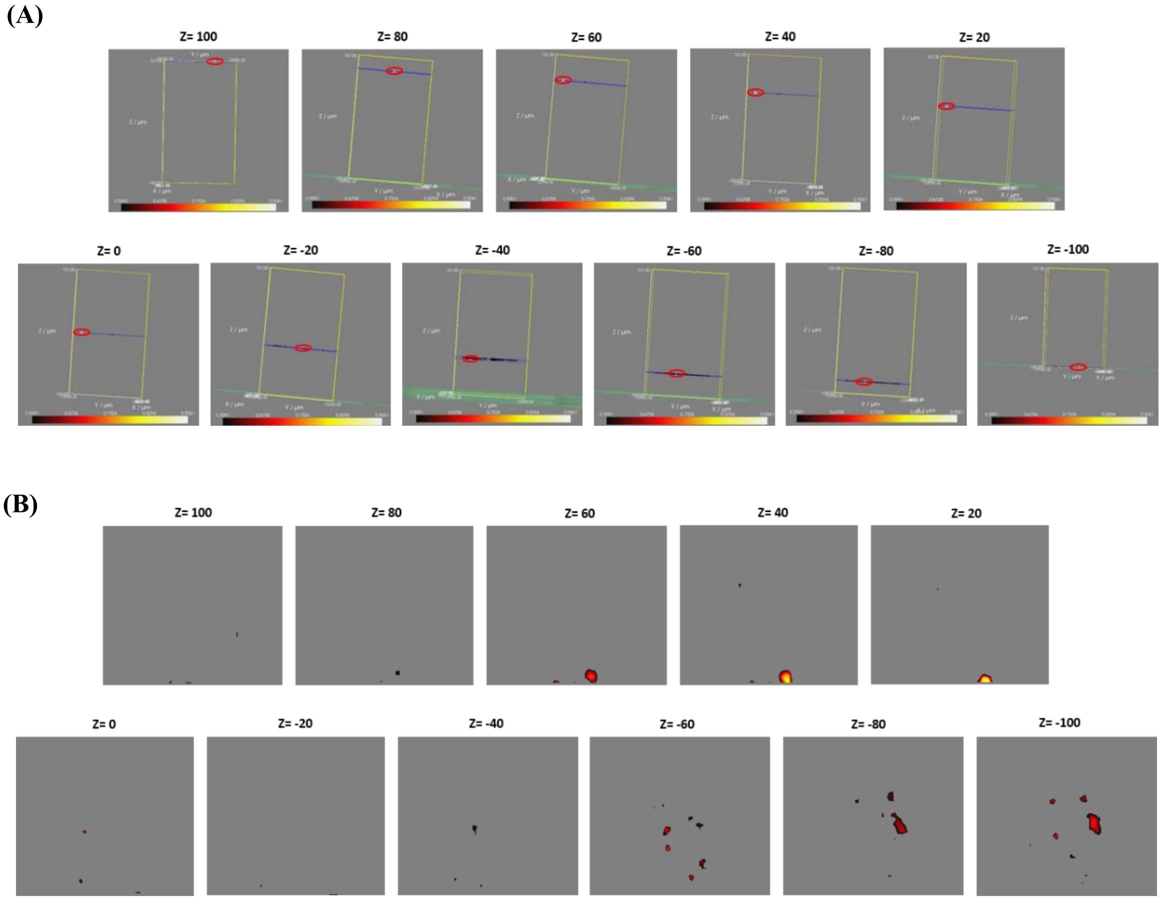

SI Figure S7: (A) 3D Raman mapping results and SERS spectra obtained at different z-axis points (red circle) through MCF-7 spheroids with ERα-AuNP nanotags (60 pM, 2h). The z-axis moves from top to bottom of the spheroids to get the SERS signal from the nanotags throughout the spheroid. (B) Z-plane representing the different z-axis points of MCF-7 spheroids SERS mapping. The plane shows that SERS signal from ERα-AuNPs is detected in different areas within the spheroids suggesting the penetration of the nanotags into the spheroids. A 20× magnification NIR APO Nikon water immersion objective with a 0.40 NA was used on the samples at a laser power of 12 mW (100% power) at the sample, from a HeNe 633 nm excitation source with step size y,x: 3.0 µm, z: 4.0 µm, 0.1s acquisition time and a 1200 l/m grating in high confocality mode. The minimum and maximum look up table thresholds was set to exclude any poorly correlating or noisy spectra (min= 0.6).

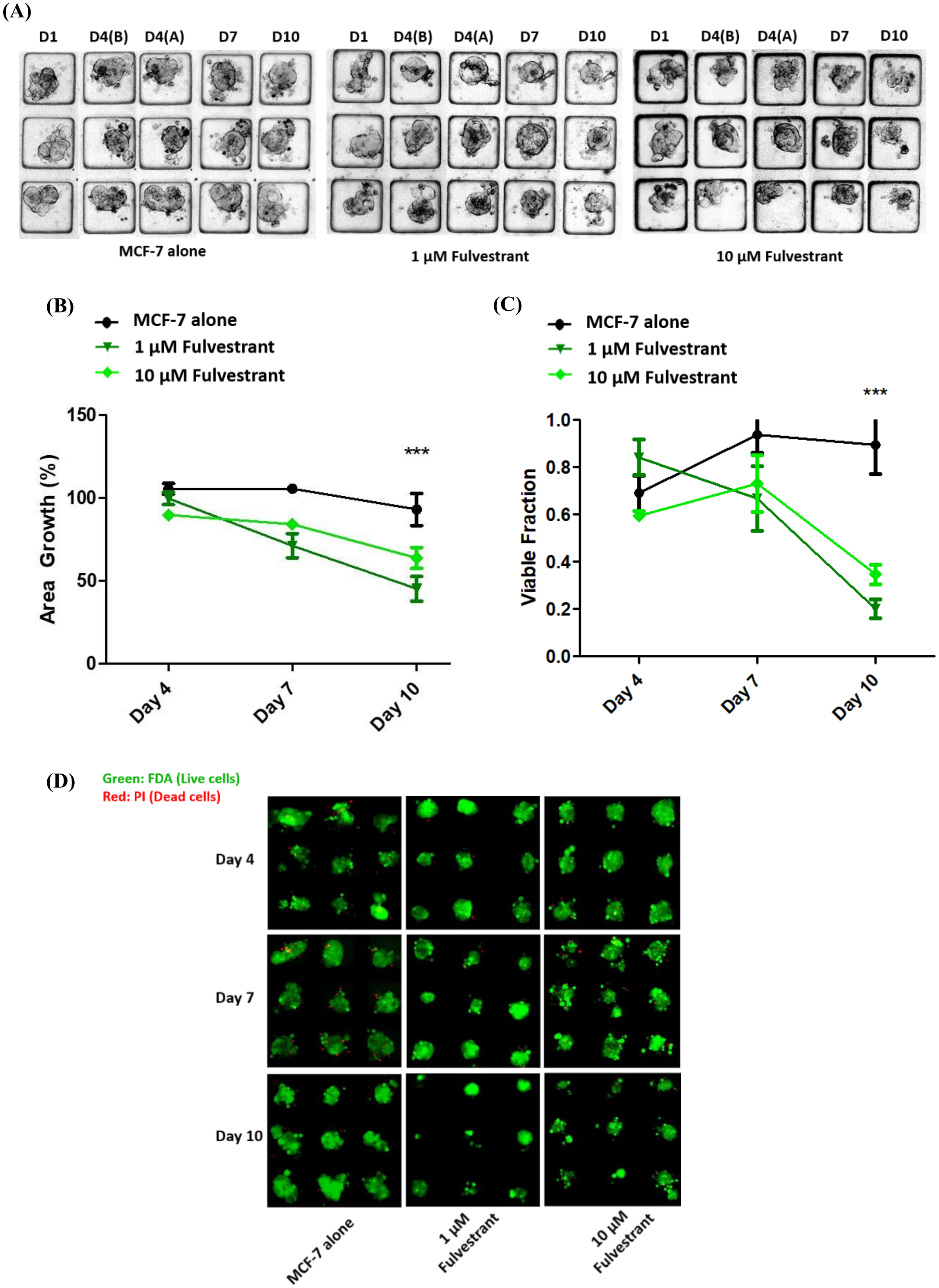

SI Figure S8: Fulvestrant treatment affected the growth and viability of MCF-7 spheroids in the microfluidic devices. (A) Brightfield imaging analysis was carried out on different days in spheroids without treatment (MCF-7 alone) and with 1 μM and 10 μM fulvestrant treatment. There was a significantly greater disaggregation with 10 μM (light green) fulvestrant treated spheroids in comparison to the untreated spheroids (black) by day 10. (B) Calculation of the percentage area growth of untreated spheroids (black), spheroids with 1 μM (dark green) and 10 μM (light green) fulvestrant treatment. Error bars presented as mean ± S.D. (C) Calculation of viable fraction of untreated spheroids (black), spheroids treated with 1 μM fulvestrant (dark green) and 10 μM fulvestrant (light green). Error bars presented as mean ± S.D (D) Representative images of spheroid viability at different time points, green is fluorescein diacetate (FDA) and red is propidium iodide (PI).

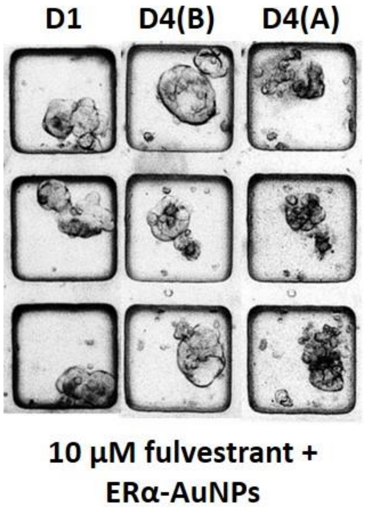

SI Figure S9: SI Figure S9: The spheroids were incubated at day 4 with ERα-AuNP nanotags after 24 h fulvestrant treatment. Bright field images of spheroids treated with 10 μM fulvestrant on day 4 before the addition of ERα-AuNP nanotags D4(B) and after the addition of ERα-AuNP nanotags D4(A). The darker colouring of the spheroid (D4(A) shows nanoparticle accumulation.
